# USP11 mediates repair of DNA–protein cross-links by deubiquitinating SPRTN metalloprotease

**DOI:** 10.1016/j.jbc.2021.100396

**Published:** 2021-02-07

**Authors:** Megan Perry, Meghan Biegert, Sai Sundeep Kollala, Halle Mallard, Grace Su, Manohar Kodavati, Natasha Kreiling, Alexander Holbrook, Gargi Ghosal

**Affiliations:** 1 Department of Genetics, Cell Biology and Anatomy, University of Nebraska Medical Center, Omaha, Nebraska, USA; 2 Department of Biology, Doane University, Crete, Nebraska, USA; 3 Department of Radiation Oncology, Houston Methodist Research Institute, Houston, Texas, USA; 4 Fred and Pamela Buffett Cancer Center, Omaha Nebraska, USA

**Keywords:** DPC, DPC repair, TOP1-ccs, SPRTN, monoubiquitinated SPRTN, deubiquitination, USP11, Co-IP, Co-Immunoprecipitations, CPT, camptothecin, DNMT1, DNA methyl transferase 1, DPC, DNA–protein cross-link, DSB, double-stranded DNA breaks, dsDNA, double-stranded DNA, DUB, deubiquitinase, HR, homologous recombination, HU, hydroxyurea, IR, ionizing radiation, MMC, mitomycin c, NER, nucleotide excision repair, RADAR, rapid approach to DNA adduct recovery, RJALS, Ruijs–Aalfs syndrome, SFB, S-FLAG-streptavidin binding peptide, SSB, single-stranded DNA breaks, ssDNA, single-stranded DNA, TAP-MS, tandem-affinity purification and mass spectrometry, TLS, translesion synthesis, TOP1, topoisomerase I, TOP2, topoisomerase II, TOP1-ccs, TOP1 cleavage complexes, TOP2-ccs, TOP2 cleavage complexes, Ub-SPRTN, monoubiquitinated SPRTN, USP, ubiquitin specific protease

## Abstract

DNA–protein cross-links (DPCs) are toxic DNA lesions that interfere with DNA metabolic processes such as replication, transcription, and recombination. USP11 deubiquitinase participates in DNA repair, but the role of USP11 in DPC repair is not known. SPRTN is a replication-coupled DNA-dependent metalloprotease that cleaves proteins cross-linked to DNA to promote DPC repair. SPRTN function is tightly regulated by a monoubiquitin switch that controls SPRTN auto-proteolysis and chromatin accessibility during DPC repair. Previously, VCPIP1 and USP7 deubiquitinases have been shown to regulate SPRTN. Here, we identify USP11 as an SPRTN deubiquitinase. USP11 interacts with SPRTN and cleaves monoubiquitinated SPRTN in cells and *in vitro*. USP11 depletion impairs SPRTN deubiquitination and promotes SPRTN auto-proteolysis in response to formaldehyde-induced DPCs. Loss of USP11 causes an accumulation of unrepaired DPCs and cellular hypersensitivity to treatment with DPC-inducing agents. Our findings show that USP11 regulates SPRTN auto-proteolysis and SPRTN-mediated DPC repair to maintain genome stability.

DNA–protein cross-links (DPCs) are irreversible covalent cross-linking of proteins to the DNA. DPCs can be generated by the action of oxygen free radicals, reactive nitrogen species, and reactive aldehydes generated as by-products of cellular respiration and metabolism or by exposure to exogenous DNA damaging agents such as UV radiation, ionizing radiation (IR), and chemotherapeutic drugs (1, 2). Covalent cross-linking of proteins to the unperturbed duplex DNA, generated by formaldehyde, IR, UV rays, and platinum-based chemotherapeutic drugs, is classified as type 1 DPCs. Trapped DNA Polβ and PARP1 at the 5′ and 3′ ends of single-stranded DNA breaks (SSBs), respectively, represent type 2 DPCs. Type 3 and type 4 DPCs arise from abortive topoisomerase–DNA enzymatic reactions, resulting in the cross-linking of topoisomerase I (TOP1) to the 3′ end of an SSB or topoisomerase II (TOP2) to the two 5′ ends of a double-stranded DNA break (DSB) (3). Cross-linking of specific DNA metabolizing enzymes, such as TOP1 and TOP2, DNA polymerase, and DNA methyl transferase (DNMT1), is also known as enzymatic DPCs (4). Irrespective of the type or source of the lesion, all DPCs are steric blockades that disrupt DNA replication, transcription, recombination, and repair processes. Unrepaired or misrepaired DPCs lead to genome instability, resulting in tumorigenesis and genetic diseases (4, 5).

Given the different types of DPCs and myriad of agents that generate them, the precise molecular mechanism underlying the DPC repair pathway has remained elusive. Genetic and biochemical experiments in different model organisms have suggested the nucleotide excision repair (NER) and homologous recombination (HR) pathways mitigate the genotoxic effects of DPCs (6, 7, 8, 9). The repair of TOP1 cleavage complexes (TOP1-ccs) and TOP2 cleavage complexes (TOP2-ccs) has been widely investigated. TOP1-ccs and TOP2-ccs are enzymatic DPCs repaired by tyrosyl-DNA phosphodiesterases TDP1 and TDP2, respectively. TDP1 and TDP2 directly hydrolyze the phosphotyrosyl bond between the protein and DNA in the DPC. Following DPC removal, the DNA breaks are subsequently repaired by SSB, HR, or nonhomologous end joining repair pathways (3, 10). In addition to TDP1, APEX2 in human and Apn2 in yeast have been shown to remove TOP1-ccs (11, 12). The proteasome also aids the removal of TOP1-ccs, TOP2-ccs, Polβ cross-linked to DNA, and DPCs generated by formaldehyde. However, polyubiquitination of DPCs was observed only in TOP1-ccs and TOP2-ccs, not in formaldehyde-induced DPCs (13, 14). Studies in *Xenopus* egg extracts showed that when the replisome collides with DPCs, the CMG helicase stalls and the DPC is proteolyzed into a peptide–DNA adduct that is bypassed by translesion synthesis (TLS) polymerases, but proteasome inhibition had no significant effect on DPC repair (15). Concurrently, yeast Wss1 was identified as a DNA-dependent metalloprotease that cleaves both enzymatic TOP1-ccs and nonenzymatic formaldehyde-induced DPCs during S-phase (16). Subsequently, SPRTN protease was shown to repair DPCs in mammalian cells (17, 18, 19). Recently, Ddi1 aspartic protease was identified in yeast and was shown to repair DPCs independent of the 20S proteasome (20). Collectively, these studies suggest that DPCs are degraded and removed by a repair pathway that is dependent on either the proteasome or a specific protease.

SPRTN (also known as DVC1/C1orf124), the mammalian functional homolog of yeast Wss1, is a replication-coupled DNA-dependent metalloprotease (17, 18). SPRTN was initially identified as a protein required for repair of UV-induced DNA lesions, restart of stalled DNA replication forks, and as a regulator of TLS (21, 22, 23, 24, 25, 26, 27). SPRTN associates with the DNA replication machinery and loss of SPRTN impaired replication fork progression (18). SPRTN protease activity is mediated by the SprT domain of SPRTN, which contains the HEXXH catalytic motif. Like Wss1, SPRTN protease cleaves TOP1, TOP2, histone H1, H2A, H2B, H3, and HMG1 in the presence of single-stranded DNA (ssDNA) (17, 18). SPRTN also drives auto-proteolysis *in trans* in the presence of ssDNA and double-stranded DNA (dsDNA) (28). Crystal structure of the SprT domain revealed a metalloprotease subdomain and Zn^2+^-binding subdomain, which regulate ssDNA binding and protease activity of SPRTN (28). SPRTN depletion sensitized cells to treatment with formaldehyde and etoposide, suggesting a role of SPRTN in the repair of formaldehyde-induced DPCs and TOP2-ccs, respectively (17, 18, 19). In humans, biallelic mutations in *SPRTN* lead to Ruijs–Aalfs syndrome (RJALS) characterized by genome instability, segmental progeria, and early-onset hepatocellular carcinoma. RJALS patient cells were defective in SPRTN protease activity, displayed defects in replication fork progression and hypersensitivity to DPC-inducing agents (29). Loss of *Sprtn* in mice resulted in embryonic lethality, while *Sprtn* hypomorphic mice recapitulated some of the progeroid phenotypes and developed spontaneous tumorigenesis in the liver with increased accumulation of DPCs in the liver tissue. Mouse embryonic fibroblasts from *Sprtn* hypomorphic mice displayed accumulation of unrepaired TOP1-ccs and were hypersensitive to treatment with DPC-inducing agents (30, 31). These studies showed that SPRTN metalloprotease repairs replication-coupled DPCs in the genome, thereby protecting cells from DPC-induced genome instability, cancer, and aging.

A recent study performed in *Xenopus* egg extracts showed that both SPRTN and the proteasome can repair replication-coupled DPCs but are activated by distinct mechanisms. The recruitment of the proteasome to DPCs required DPC polyubiquitination, while SPRTN was able to degrade nonubiquitinated DPCs. SPRTN-mediated DPC proteolysis depended on the extension of the nascent DNA strand to within a few nucleotides of the DPC lesion, indicating that polymerase stalling at a DPC on either the leading or lagging strand activates SPRTN. SPRTN depletion impaired TLS following DPC proteolysis in both proteasome-mediated and SPRTN-mediated replication-coupled DPC repair, suggesting that in addition to DPC proteolysis, SPRTN regulates bypass of peptide–DNA adducts by TLS during DNA replication (32).

SPRTN is a sequence-nonspecific protease that predominantly cleaves substrates in unstructured regions in the vicinity of lysine, arginine, and serine residues (17, 18). Several mechanisms regulate SPRTN function in DPC repair. SPRTN protease activity is stimulated by DNA binding, while posttranslational modification of SPRTN governs both its protease activity and recruitment to the DPC on chromatin. CHK1 kinase phosphorylates SPRTN at the C-terminal (S373, S374, and S383) and enhances SPRTN protease activity and recruitment to chromatin (33). SPRTN is also monoubiquitinated, which prevents SPRTN access to chromatin and regulates SPRTN protein levels (17, 23, 34, 35). Upon DPC induction, ATM/ATR kinase activates VCPIP1/VCIP135 deubiquitinase, which in turn deubiquitinates SPRTN, regulating its chromatin localization. Deubiquitination of SPRTN is a prerequisite for its subsequent acetylation, which promotes SPRTN relocation to chromatin (35). In contrast, a recent study showed that monoubiquitination of SPRTN does not regulate SPRTN access to chromatin, but instead, promotes SPRTN auto-proteolysis *in trans* while also priming SPRTN for proteasomal degradation in *cis*. USP7 deubiquitinase deubiquitinates SPRTN upon DPC induction, antagonizing the autocatalytic cleavage and subsequent inactivation of SPRTN (34). Here, we have identified USP11 as a ubiquitin protease that regulates SPRTN monoubiquitination and auto-proteolysis upon DPC induction.

USP11 (ubiquitin carboxyl-terminal hydrolase or ubiquitin-specific protease 11) belongs to the ubiquitin-specific protease (USP or UBP) family of deubiquitinases (DUBs). USP11 participates in processes such as TGFβ signaling, proinflammatory signaling, viral replication, and NF-κB signaling by regulating the protein stability of various targets such as ARID1A, TβRII, CDKN2A, RAE1, XIAP, HPV16-E7, and IκBα (36, 37, 38, 39, 40, 41). USP11 also functions in DSB repair, wherein USP11 deubiquitinates H2AX to regulate the recruitment of RAD51 and 53BP1 to damage foci (42, 43). USP11 deubiquitinates PALB2 and promotes BRCA1-PALB2-BRCA2 complex formation (44).

USP11 confers cellular resistance to PARP1 inhibitors that trap PARP1 to DNA (45). However, the function of USP11 in DPC repair is not reported. In this study, we show that USP11 is a novel interactor of SPRTN and deubiquitinates SPRTN in cells and *in vitro*. Depletion of USP11 leads to increased SPRTN auto-proteolysis, an accumulation of unrepaired DPCs, and sensitizes cells to DPC-inducing agents. USP11 cleaves the monoubiquitin on SPRTN upon DPC induction and regulates SPRTN-mediated DPC repair.

## Results

### USP11 is an SPRTN interacting protein

**Figure 1:**
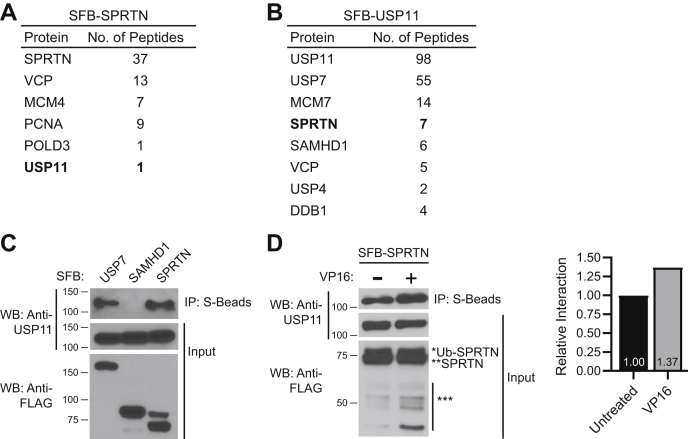
**USP11 is an SPRTN interacting protein.***A* and *B*, TAP-MS was performed using HEK 293T cells stably expressing SFB- (*A*) SPRTN or (*B*) USP11. SFB-USP11 cells were treated with 10 μM CPT for 2 h. Selected results from MS analysis (Tables S1 and S2) are shown. MS data have been deposited to the ProteomeXchange Consortium with the data set identifier PXD019923. *C*, USP11 interacts with SPRTN. HEK 293T cells were transfected with SFB-USP7, SAMHD1, or SPRTN. *D*, USP11 interacts with SPRTN in the absence and presence of DPCs. *Left panel*, HEK 293T cells transfected with SFB-SPRTN were left untreated or treated with 20 μM VP16 for 4 h. *Right panel*, graph shows quantification of USP11 interaction relative to untreated sample and normalized to the USP11 input WB. *C* and *D*, cell lysates were immunoprecipitated with S-protein agarose beads, and immunoblotting was performed using the indicated antibodies. WB, western blot. ∗∗∗ indicates SPRTN auto-cleavage products. See also Fig. S1.

Monoubiquitinated SPRTN is deubiquitinated upon DNA damage by DPC-inducing agents (17). Therefore, ubiquitination and deubiquitination of SPRTN are critical for the recruitment of SPRTN to DPC lesions. To identify SPRTN modifiers, we performed tandem-affinity purification and mass spectrometry (TAP-MS) analysis of SFB (S-FLAG-streptavidin binding peptide)-tagged SPRTN expressed in HEK 293T cells. Our MS analysis revealed several known SPRTN interactors, namely PCNA, POLD3, and VCP (Fig. 1*A* and Table S1). We screened the MS list for proteins that are known to function as a deubiquitinase and identified only one potential SPRTN deubiquitinase, USP11. Similar to POLD3, only one peptide of USP11 was immunoprecipitated in SPRTN MS analysis (Table S2). A reciprocal TAP-MS analysis of SFB-USP11 expressed in HEK 293T cells immunoprecipitated SPRTN in addition to USP7, which is a known USP11 interactor (Fig. 1*B* and Table S3). We next confirmed SPRTN-USP11 interaction by immunoprecipitation in HEK 293T cells expressing either SFB-USP7, SAMHD1, or SPRTN. USP7 is a known interactor of USP11, while SAMHD1 and SPRTN were identified in SFB-USP11 TAP-MS analysis (Fig. 1*B*). Endogenous USP11 was immunoprecipitated with SFB-USP7 and SPRTN, but not with SAMHD1 (Fig. 1*C*). We observed a slight increase in SPRTN-USP11 interaction following treatment with etoposide (VP16), a TOP2 crosslinking agent (Fig. 1*D*, Fig. S1), camptothecin (CPT), TOP1 cross-linking agent, formaldehyde (nonspecific cross-linking agent), and hydroxyurea (non-cross-linking agent) (Fig. S1).

**Figure 2:**
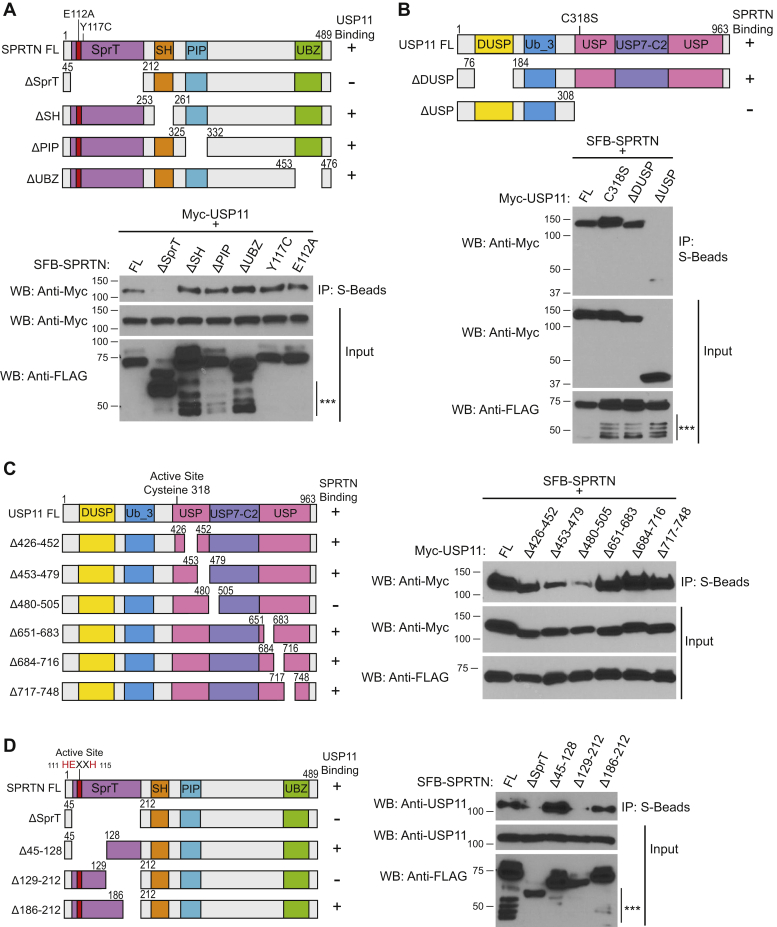
**SprT domain of SPRTN and USP domain of USP11 mediate SPRTN-USP11 interaction.***A*, *Top panel*, protein schematic of SPRTN FL, domain deletion, and point mutants. *Bottom panel*, HEK 293T cells were transfected with Myc-USP11 and SFB-SPRTN FL, deletion, or point mutants. *B*, *Top panel*, schematic of USP11 FL and deletion mutants. USP11 domains derived from Pfam predicted motifs. *Bottom panel*, HEK 293T cells were transfected with SFB-SPRTN and Myc-USP11 FL, deletion, or point mutants. *C*, *Left panel*, protein schematic of USP11 FL and USP domain deletion mutants. *Right panel*, HEK 293T cells were transfected with SFB-SPRTN and Myc-USP11 FL or USP domain deletion mutants. *D*, *Left panel*, protein schematic of SPRTN FL and SprT domain deletion mutants. *Right panel*, HEK 293T cells were transfected with SFB-SPRTN FL and SprT domain deletion mutants. *A*–*D*, cell lysates were immunoprecipitated with S-protein agarose beads, and immunoblotting was performed using the indicated antibodies. WB, western blot. ∗∗∗ indicates SPRTN auto-cleavage products.

Both SPRTN and USP11 are multidomain proteins. To map interaction sites on SPRTN and USP11, we generated domain deletion mutant constructs of SPRTN (ΔSprT, ΔSH, ΔPIP, and ΔUBZ) and USP11 (ΔDUSP and ΔUSP). Co-immunoprecipitation (Co-IP) of Myc-USP11 with SFB-SPRTN full-length (FL) or ΔSprT, ΔSH, ΔPIP, ΔUBZ, E112A catalytic inactive, and Y117C (SPRTN mutation identified in RJALS patients) mutant constructs showed that SPRTN-USP11 interaction was lost with deletion of the N-terminal SprT domain of SPRTN (Fig. 2*A*). Co-IP experiments of SFB-SPRTN with Myc-USP11 FL, C318S catalytic inactive, ΔDUSP, and ΔUSP mutant constructs showed that SPRTN interacts with the C-terminal USP domain of USP11 (Fig. 2*B*). Because the USP domain of USP11 is 654 aa long, we generated several internal deletion constructs within the USP domain. Co-IP experiments of SFB-SPRTN with Myc-USP11 FL and internal deletion constructs of the USP domain showed that the SPRTN interaction site resides within the 480 to 505 aa region of USP11 (Fig. 2*C*). Similarly, we generated internal deletion mutants within the SprT domain of SPRTN to map the site of USP11 interaction in SPRTN. We found that deletion of the SprT domain and 129 to 212 aa region in SPRTN abrogated USP11 binding. However, deletion of 186 to 212 aa fragment in SPRTN retained USP11 interaction, indicating that the USP11 binding site lies within 129 to 186 aa region of SPRTN (Fig. 2*D*).

### USP11 deubiquitinates SPRTN in cells and *in vitro*

**Figure 3:**
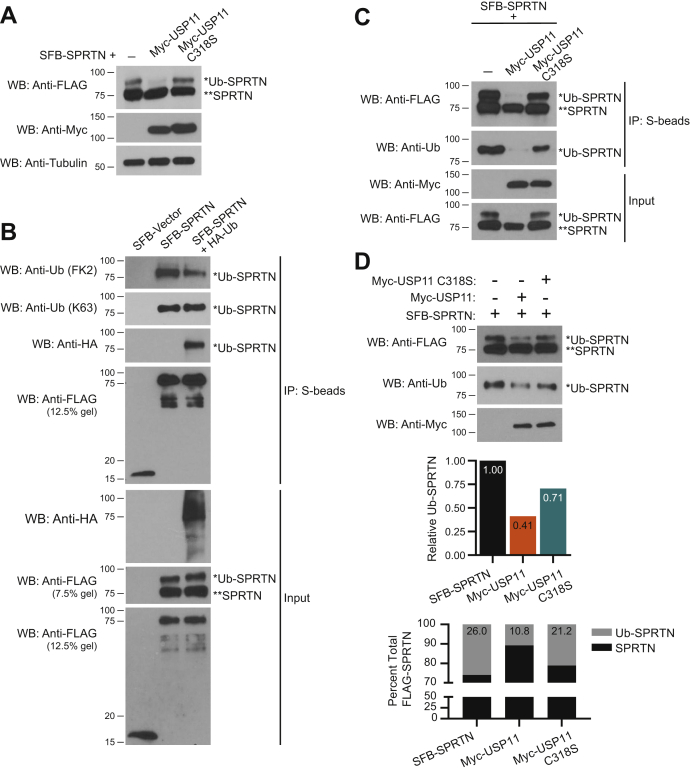
**USP11 deubiquitinates SPRTN in cells and *in vitro*.***A*, HEK 293T cells were transfected with SFB-SPRTN and Myc-USP11 FL or C318S. Cell lysates were immunoblotted with the indicated antibodies. *B*, HEK 293T cells were transfected with SFB-empty vector, SFB-SPRTN, or SFB-SPRTN and HA-Ubiquitin. *C*, HEK 293T cells were transfected with SFB-SPRTN and Myc-USP11 FL or C318S mutant. *B* and *C*, cell lysates were immunoprecipitated with S-protein agarose beads and immunoblotted with indicated antibodies. *D*, USP11 deubiquitinates SPRTN *in vitro*. *Top panel*, SFB-SPRTN, Myc-USP11, and Myc-USP11 C318S were purified as described in experimental procedures. SFB-SPRTN was incubated with Myc-USP11 or Myc-USP11 C318S mutant proteins overnight at 30 °C. The reaction mixture was immunoblotted with indicated antibodies (*C* and *D*, anti-Ub blots were probed with K63 ubiquitin antibody). *Middle panel*, graph shows relative Ub-SPRTN values quantified from anti-Ubiquitin WB. *Bottom panel*, graph shows percent total FLAG-SPRTN. Percent of unmodified and monoubiquitinated SPRTN was calculated from anti-FLAG WB. WB, western blot. See also Fig. S2.

Based on our observation that SPRTN and USP11 interact (Figs. 1 and 2) and because USP11 is a deubiquitinase (36, 37, 44), we investigated whether SPRTN is a substrate for USP11 deubiquitinase. Indeed, we found that overexpression of Myc-USP11 FL, but not C318S catalytic inactive mutant was able to deubiquitinate SFB-SPRTN in HEK 293T cells (Fig. 3*A*). Previous studies have shown that SPRTN is monoubiquitinated (17, 23). To confirm monoubiquitination of SFB-SPRTN, we transfected SFB-tagged vector, SPRTN or SPRTN and HA-ubiquitin (HA-Ub) in HEK 293T cells, immunoprecipitated and immunoblotted with FLAG, HA, and ubiquitin (FK2 and K63-Ub) antibodies. We observed that the FK2 antibody, which recognizes both monoubiquitin and polyubiquitin chains, probed the modified band of SPRTN (Ub-SPRTN). Notably, FK2 antibody recognized only a single modified band indicating that the modification on SPRTN corresponds to monoubiquitination (Fig. 3*B*, anti-Ub FK2 IP blot). We also found that K63-Ub antibody recognized only the single monoubiquitin band and no K63-linked Ub-chains were observed (Fig. 3*B*, anti-Ub K63 IP blot). FK2 and K63-Ub antibodies probed the modification on SPRTN in SFB-SPRTN and SFB-SPRTN-HA-Ub and not in SFB-vector immunoprecipitated samples. Notably, SPRTN modified by HA-Ub was recognized by HA antibody in SFB-SPRTN-HA-Ub, but not in SFB-vector or SFB-SPRTN immunoprecipitations confirming that the modification on SPRTN corresponds to ubiquitination. Similar to FK2 and K63-Ub antibodies, HA antibody probed a single HA-Ub band and no polyubiquitination was observed (Fig. 3*B*, anti-HA IP blot). These observations indicate that the modification on SPRTN corresponds to monoubiquitination.

We next confirmed deubiquitination of monoubiquitinated SPRTN by performing deubiquitination assays in HEK 293T cells expressing SFB-SPRTN either alone or in combination with Myc-USP11 FL or C318S mutant (Fig. 3*C*) and SFB-SPRTN and HA-Ub constructs expressed either alone or in combination with Myc-USP11 FL and C318S mutant (Fig. S2*A*). SFB-SPRTN was immunoprecipitated and monoubiquitination of SPRTN was examined by immunoblotting with Ub (Fig. 3*C*) or HA antibodies (Fig. S2*A*). We observed that monoubiquitinated SPRTN is deubiquitinated by USP11 FL, but not C318S deubiquitinase inactive mutant (Fig. 3*C* and Fig. S2*A*). To show that deubiquitination of SPRTN by USP11 is direct, we purified SFB-SPRTN, Myc-USP11, and Myc-USP11 C318S proteins from HEK 293T cells and performed an *in vitro* DUB reaction by incubating SFB-SPRTN alone or with Myc-USP11 or Myc-USP11 C318S purified proteins. Deubiquitination of SPRTN was examined by immunoblotting with ubiquitin antibody (Fig. 3*D*). We observed that monoubiquitination of SPRTN was dramatically reduced in USP11 FL compared with USP11 C318S mutant (Fig. 3*D*). We further corroborated this observation in a similar *in vitro* DUB reaction by incubating SFB-HA-Ub-SPRTN alone or with Myc-USP11 or Myc-USP11 C318S purified proteins and immunoblotted for HA-Ub (Fig. S2*B*). We observed a marginal reduction in monoubiquitinated SPRTN with USP11 C318S mutant (Fig. 3*D* and Fig. S2*B*), which could be due to only partial loss of USP11 deubiquitinase activity, as the catalytic core of all UBP family DUBs is comprised of both a Cys (nucleophile) and His (proton acceptor) residue (46). Together these findings demonstrate that USP11 deubiquitinates monoubiquitinated SPRTN in cells and *in vitro*.

### USP11 is required for cell survival upon DNA damage by DPC-inducing agents

**Figure 4:**
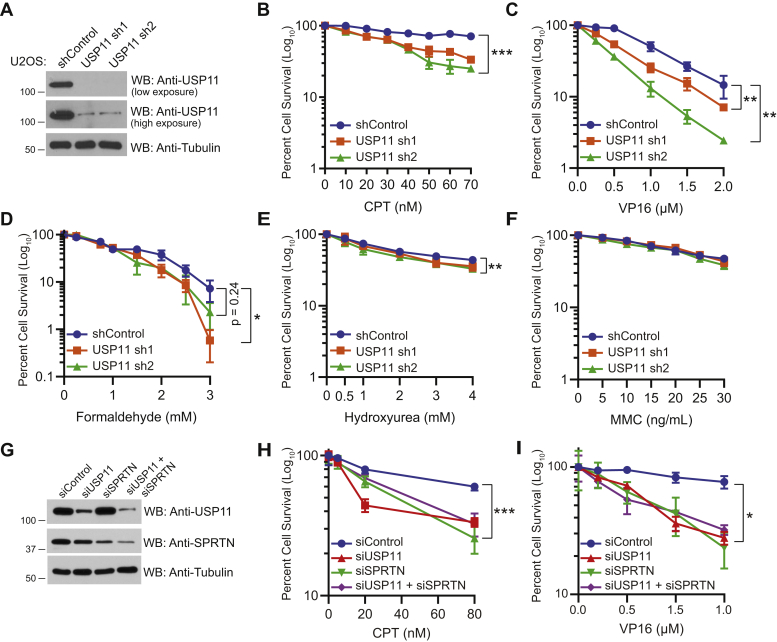
**USP11 is required for cell survival upon DNA damage by DPC-inducing agents.***A*, WB showing knockdown efficiency of USP11 in U2OS cells stably expressing nonsilencing shRNA control or two different shRNA sequences (sh1 and sh2) targeted to USP11. *B*–*F*, clonogenic cell survival curves for shRNA control and USP11 knockdown U2OS treated with indicated concentrations of (*B*) CPT for 24 h, (*C*) VP16 for 4 h, (*D*) formaldehyde for 20 min, (*E*) HU for 24 h, or (*F*) MMC for 24 h. *G*, WB showing knockdown efficiency of USP11 and SPRTN. *H* and *I*, clonogenic cell survival curves for USP11 and SPRTN single and double -knockdown cells treated with indicated concentrations of (*H*) CPT for 24 h or (*I*) VP16 for 2 h. *B*–*F*, *H* and *I*, percent cell survival was calculated. Data are presented as mean ± SD (n = 3). Statistical analysis: Two-tailed paired *t*-test was performed using confidence interval =90% and α = 0.1; ∗*p* ≤ 0.1, ∗∗*p* ≤ 0.05, ∗∗∗*p* ≤ 0.01. WB, western blot. See also Fig. S3.

SPRTN is required for repair of DPCs (16, 18). However, the function of USP11 in response to DPC lesions and DPC repair is not known. To gain insight into the functional relevance of SPRTN-USP11 interaction, we first examined whether USP11 functions in response to DNA damage caused by DPC-inducing agents. We generated USP11 knockdown in U2OS (Fig. 4*A*) and A549 (Fig. S3*A*) cells to rule out cell type specificity effects in the experiments. Negative control and USP11 knockdown U2OS and A549 cells were untreated or treated with increasing concentrations of DPC-inducing agents, and clonogenic cell survival was examined 10 days posttreatment (Fig. 4 and Fig. S3). We found that depletion of USP11 sensitized U2OS cells to treatment with CPT (Fig. 4*B*), VP16 (Fig. 4*C*), and formaldehyde (Fig. 4*D*). We observed mild sensitivity of USP11 knockdown cells to hydroxyurea (HU) treatment, which causes stalled DNA replication forks due to depletion of dNTP pools (Fig. 4*E*). Importantly, we observed no sensitivity of USP11 knockdown cells compared with the negative control to treatment with mitomycin c (MMC), which generates interstrand DNA cross-links (Fig. 4*F*). Similarly, USP11 knockdown in A549 cells sensitized cells to CPT (Fig. S3*B*), VP16 (Fig. S3*C*), and formaldehyde (Fig. S3*D*) treatment. USP11 knockdown A549 cells displayed mild sensitivity to HU (Fig. S3*E*) and no sensitivity to MMC (Fig. S3*F*) treatment. These observations suggest that USP11 deubiquitinase is required for cell survival upon treatment with DNA–protein, but not DNA–DNA cross-linking agents. To determine whether USP11 functions in the SPRTN-mediated DPC repair pathway, we performed clonogenic cell survival assays in USP11 and SPRTN single or double-knockdown cells (Fig. 4*G*) treated with CPT (Fig. 4*H*) and VP16 (Fig. 4*I*). We observed that USP11 and SPRTN single or double-knockdown cells displayed similar hypersensitivity upon treatment with CPT (Fig. 4*H*) and VP16 (Fig. 4*I*) compared with the negative control. Further, SPRTN overexpression (Fig. S3*G*) rescued the sensitivity of USP11 knockdown cells to CPT (Fig. S3*H*), VP16 (Fig. S3*I*), and formaldehyde (Fig. S3*J*) treatment. Collectively, the data suggests that USP11 functions in SPRTN-mediated DPC repair.

### USP11 deubiquitinates SPRTN upon DPC induction

Our data shows that SPRTN is a substrate of USP11 deubiquitinase (Fig. 3). To examine the effect of USP11 on SPRTN protein stability, we expressed SFB-SPRTN E112A in combination with increasing concentrations of either Myc-USP11 or Myc-USP11 C318S mutant. SPRTN E112A mutant was used to prevent changes in SPRTN protein levels mediated by auto-proteolysis. Increasing concentrations of USP11, but not USP11 C318S, led to a reduction in monoubiquitinated SPRTN (Fig. S4*A*). Importantly, SPRTN levels remained unchanged with increasing concentration of USP11 C318S deubiquitinase inactive mutant, suggesting that USP11 does not regulate the stability of SPRTN (Fig. S4*A*). Further, we observed a decrease in endogenous unmodified SPRTN levels with a subsequent increase in Ub-SPRTN in USP11 knockdown cells compared with negative control (Fig. S4*B*), indicating that USP11 deubiquitinates monoubiquitinated SPRTN.

**Figure 5:**
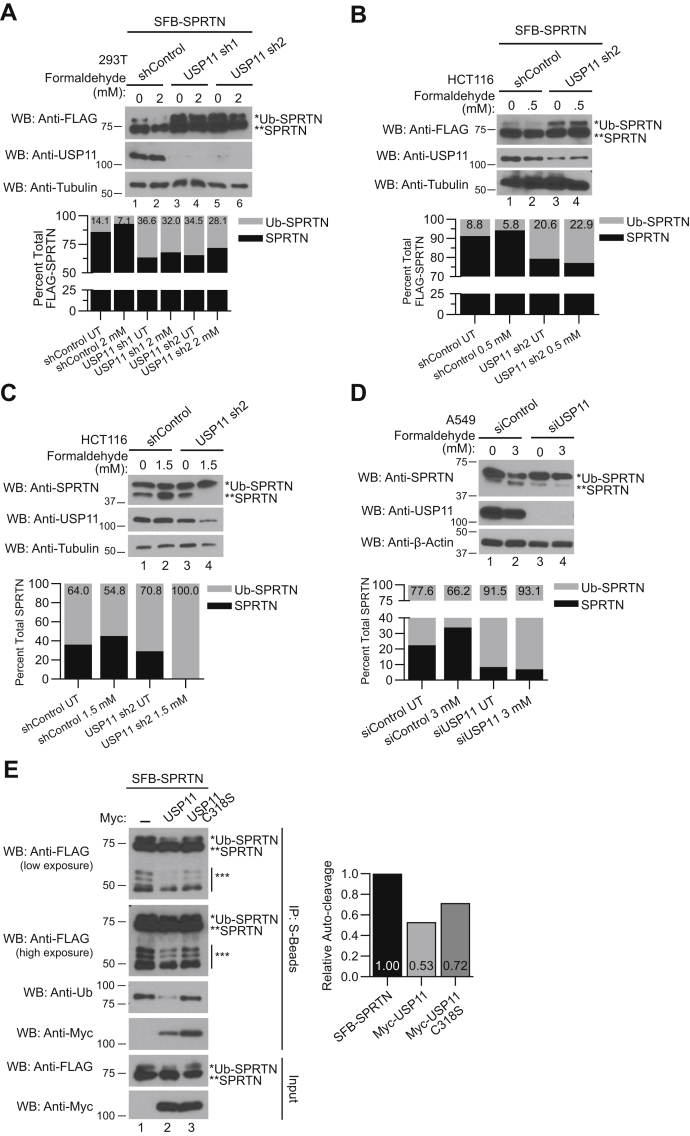
**USP11 deubiquitinates SPRTN upon DPC induction.***A* and *B*, HEK 293T (*A*) or HCT116 (*B*) cells stably expressing shRNA targeted to USP11 were transfected with SFB-SPRTN. Twenty-four hours later, cells were treated with the indicated concentrations of formaldehyde for 2 h. *C*, HCT116 cells stably expressing shRNA targeted to USP11 were treated with the indicated concentrations of formaldehyde for 1 h. The nuclear fraction was isolated and used for WB. *D*, A549 cells were transfected with siRNA targeted to USP11. Seventy-two hours later, cells were treated with the indicated concentrations of formaldehyde for 2 h. *A*–*D*, *Top panel*, cell lysates were immunoblotted with the indicated antibodies. *Bottom panel*, graph shows percent total FLAG-SPRTN or percent total SPRTN quantified from anti-FLAG or anti-SPRTN blots, respectively. The percent of unmodified and monoubiquitinated SPRTN was calculated. *E*, HEK 293T cells were transfected with SFB-SPRTN alone or in combination with Myc-USP11 FL or C318S mutant. *Left panel*, cell lysates were immunoprecipitated with S-protein agarose beads, and immunoblotting was performed using the indicated antibodies. Anti-Ub blot was probed with K63 ubiquitin antibody. *Right panel*, graph showing quantification of SFB-SPRTN auto-cleavage products. Anti-FLAG high exposure blot was used for quantification. WB, western blot. See also Figs. S4, S5, and S6.

A previous study showed that upon DNA damage by DPC-inducing agents, monoubiquitinated SPRTN is deubiquitinated and localized to chromatin (17). We examined deubiquitination of SFB-SPRTN and SFB-SPRTN E112A catalytic mutant in HEK 293T cells upon treatment with formaldehyde. We observed a stepwise reduction in monoubiquitinated SPRTN FL (Fig. S5*A*) and SPRTN E112A (Fig. S5*B*) levels with increasing concentrations of formaldehyde. Similarly, we observed deubiquitination of SPRTN upon CPT (Fig. S5*C*) and VP16 (Fig. S5*D*) treatment, but at very high concentrations (≥750 μM). Notably, cell viability was decreased at such high concentrations. The inability to detect robust SPRTN deubiquitination by western blotting upon treatment with low concentrations of CPT and VP16 could be due to the action of these drugs on cross-linking specific enzymes, TOP1 and TOP2, respectively. In contrast, formaldehyde treatment generates greater numbers and diverse types of DPCs, thus generating a robust SPRTN deubiquitination response at low concentration and treatment times. We next asked if USP11 is required for SPRTN deubiquitination in response to DPCs. To address this question, we introduced SFB-SPRTN into negative control and USP11 knockdown HEK 293T (Fig. 5*A*) and HCT116 cell lines (Fig. 5*B*) and observed monoubiquitinated SPRTN levels in the absence and presence of DPCs generated by formaldehyde treatment. Monoubiquitinated SPRTN levels were increased in the absence of USP11 compared with the negative control in both HEK 293T (Fig. 5*A*, lanes 1, 3 and 5) and HCT116 (Fig. 5*B*, lanes 1 and 3) untreated cells. Importantly, we observed a reduction in SFB-SPRTN deubiquitination in USP11 knockdown HEK 293T (Fig. 5*A*, lanes 4 and 6) compared with the negative control (Fig. 5*A*, lane 2) upon formaldehyde treatment. Similarly, USP11 depletion in HCT116 inhibited SFB-SPRTN deubiquitination (Fig. 5*B*, lane 4) compared with negative control (Fig. 5*B*, lane 2) upon formaldehyde treatment, suggesting that USP11 depletion inhibits SPRTN deubiquitination upon DPC induction. We next examined deubiquitination of endogenous SPRTN in the absence and presence of DPCs in USP11 knockdown HCT116 and A549 cells. USP11 depletion led to an increase in monoubiquitinated SPRTN levels compared with the negative control in the absence of damage in HCT116 (Fig. 5*C*, lanes 1 and 3) and A549 (Fig. 5*D*, lanes 1 and 3) cells. Notably, SPRTN deubiquitination was abrogated in USP11 knockdown HCT116 (Fig. 5*C*, lane 4) and A549 (Fig. 5*D*, lane 4) cells upon treatment with formaldehyde. Collectively, these findings demonstrate that USP11 is required for SPRTN deubiquitination upon DPC induction.

A previous study showed that VCPIP1 deubiquitinates SPRTN, promoting SPRTN localization to chromatin (35). Therefore, we examined SPRTN chromatin localization in USP11 and VCPIP1 single or double-knockdown cells in the absence and presence of formaldehyde treatment (Fig. S6*A*). We observed that upon treatment with formaldehyde, SPRTN is deubiquitinated (Fig. S6*A*, lane 2, total and soluble blot panels) and the unmodified SPRTN is enriched on chromatin (Fig. S6*A*, lane 2, chromatin blot panel) in negative control cells. Importantly, deubiquitination of SPRTN was abrogated in USP11 and VCPIP1 single and double-knockdown cells upon formaldehyde treatment (Fig. S6*A*, lanes 4, 6, and 8 total and soluble blot panels). However, an enrichment of unmodified SPRTN was observed on chromatin in USP11 and VCPIP1 single and double-knockdown cells (Fig. S6*A*, lanes 4, 6, and 8 chromatin blot panels). These observations show that depletion of USP11 and VCPIP1 inhibits SPRTN deubiquitination, but in contrast to previous reports, monoubiquitination does not restrict SPRTN access to chromatin.

A recent study also showed that monoubiquitination does not control SPRTN chromatin access, but instead inactivates SPRTN by triggering auto-proteolysis and priming SPRTN for polyubiquitination and degradation by the proteasome. Upon DPC induction, SPRTN is deubiquitinated by USP7, promoting SPRTN stability (34). We next examined SPRTN monoubiquitination and auto-cleavage products in cells expressing USP11 or USP11 C318S catalytic inactive mutant. We observed that USP11, but not USP11 C318S catalytic mutant, deubiquitinated SPRTN (Fig. 5*E*, anti-Ub blot) and reduced SPRTN auto-cleavage products (Fig. 5*E*, IP blot). A previous study showed that USP11 and USP7 interact, and this interaction is lost in USP7 D164AW165A and USP11 S687A mutants (47). Therefore, we asked whether USP11 and USP7 function together in regulating SPRTN auto-proteolysis in the absence and upon formaldehyde treatment. We observed that SPRTN is not deubiquitinated in USP11 and USP7 single and double-knockdown cells upon treatment with formaldehyde (Fig. S6*B*, lanes 4, 6, and 8). SPRTN auto-cleavage was reduced upon DPC induction (Fig. S6*B*), in line with the previous study (34). Importantly, SPRTN auto-cleavage products increased in USP11 (Fig. S6*B*, lane 3) and USP7 knockdown cells (Fig. S6*B*, lane 5) compared with the negative control. Notably, SPRTN auto-cleavage increased in USP11-USP7 double-knockdown cells (Fig. S6*B*, lane 7) compared with USP11 or USP7 single knockdown, suggesting that USP11 and USP7 may function together in regulating SPRTN auto-proteolysis.

### USP11 participates in DPC repair

**Figure 6:**
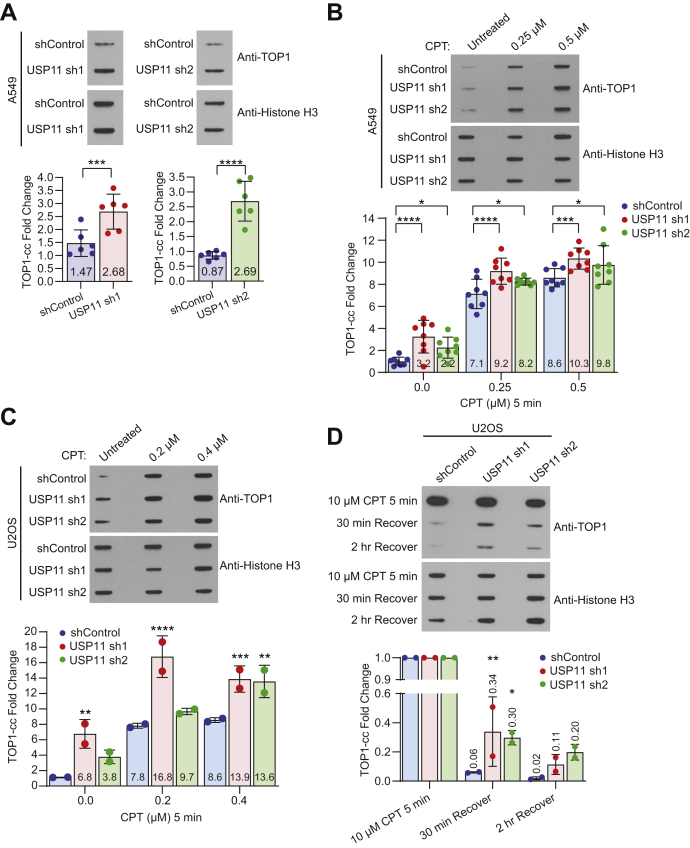
**USP11 participates in DPC repair.***A*–*D*, RADAR assays in cells stably expressing non-silencing shRNA control or two different shRNA sequences (sh1 and sh2) targeted to USP11. Sample preparation and slot blotting were completed as described in experimental procedures. *Top panel*, *A*, 500 ng of DNA isolated from untreated A549 cells was immunoblotted with TOP1 antibody to detect endogenous TOP1-ccs and histone H3 was immunoblotted as a loading control. *B*, A549 cells were treated with CPT at the indicated concentrations for 5 min. One microgram of DNA was immunoblotted with TOP1 antibody and histone H3. *C*, U2OS cells were treated with CPT at the indicated concentrations for 5 min. One microgram of DNA was immunoblotted with TOP1 antibody, and 500 ng of sample was immunoblotted with histone H3. *D*, U2OS cells were treated with 10 μM CPT for 5 min. Cells used for recovery time points were washed with PBS and left to recover in drug-free media for the indicated time. In total, 600 ng of DNA was immunoblotted with TOP1 antibody and with histone H3. *A*–*D*, *Bottom panel*, TOP1-cc fold change from respective slot blots shown above was quantified from relative abundance of TOP1 normalized to histone H3 (*A*, n = 6; C, n = 2) or normalized to amount of DNA loaded (*B*, n =8; D, n = 2). Data are presented as mean ± SD. Statistical analysis: Two-way ANOVA and Dunnett’s multiple comparison test were performed using confidence interval =90% and α = 0.1; ∗*p* ≤ 0.1, ∗∗*p* ≤ 0.05, ∗∗∗*p* ≤ 0.01, ∗∗∗∗*p* ≤ 0.001. See also Figs. S7 and S8.

Our data shows that USP11 is required for SPRTN deubiquitination (Fig. 5) and cell survival (Fig. 4) upon DPC induction, indicating that USP11 is required for DPC repair. To demonstrate that USP11 participates in DPC repair, we examined the accumulation and repair of specific DPCs in USP11 knockdown cells using a Rapid Approach to DNA Adduct Recovery (RADAR) assay. We examined TOP1-cc levels in negative control and USP11 knockdown A549 cells and found that USP11 depletion led to an increase in TOP1-ccs in the absence of DPCs induced by treatment with exogenous DPC-inducing agents (Fig. 6*A*). We next treated the negative control and USP11 knockdown A549 cells with CPT and examined TOP1-cc induction. With increasing concentration of CPT treatment, USP11 knockdown cells showed an accumulation of TOP1-ccs compared with the negative control (Fig. 6*B*). We further corroborated this finding in USP11 knockdown U2OS cells (Fig. 6*C* and Fig. S7*A*). Similar to A549 cells, USP11 knockdown in U2OS cells showed a significant increase in TOP1-cc levels compared with the negative control upon treatment with 0.4 μM CPT (Fig. 6*C*). However, knockdown of USP11 with shRNA2 resulted in only marginal increase in TOP1-cc levels at 0.2 μM CPT concentration. To rule out any variability in USP11 knockdown by shRNA, we performed RADAR assay in U2OS cells expressing USP11siRNA (Fig. S7*B*). We observed a statistically significant increase in TOP1-ccs in untreated, 0.2 μM, and 0.4 μM CPT-treated USP11 knockdown cells compared with negative control (Fig. S7*B*). We next examined DPC repair by allowing the cells to recover and repair TOP1-ccs generated from CPT treatment at high (Fig. 6*D* and Fig. S7*C*) and low (Fig. S7*D*) concentrations. We found that depletion of USP11 led to delayed TOP1-cc repair, indicated by sustained TOP1-ccs following 30 min and 2 h recovery time periods, while the majority of TOP1-ccs generated in negative control cells were repaired within 30 min (Fig. 6*D* and Fig. S7*D*) or 45 min (Fig. S7*C*), and repair was completed by the 2 h recovery time point (Fig. 6*D* and Fig. S7*C*). We examined accumulation of TOP2-cc and observed a stepwise increase in TOP2-ccs levels in USP11 knockdown cells compared with negative control with increasing concentration of VP16 treatment (Fig. S8*A*). TOP2-cc repair was delayed in USP11 knockdown cells compared with the negative control after 30 min recovery period (Fig. S8*B*). These findings demonstrate that depletion of USP11 delays DPC repair. To examine accumulation of nonspecific DPCs induced by formaldehyde treatment, we performed the ARK assay, a robust method to isolate global DPCs as described in a recent study (48). Similar to TOP1-ccs and TOP2-ccs, USP11 knockdown cells displayed increased accumulation of nonspecific DPCs compared with negative control upon treatment with formaldehyde (Fig. S8*C*). Notably, treatment with hydroxyurea, a non-cross-linking agent, did not lead to accumulation of DPCs in USP11 knockdown and negative control cells (Fig. S8*C*). Together, these observations suggest that USP11 participates in DPC repair.

## Discussion

In this study, we showed that USP11 interacts with SPRTN protease and deubiquitinates monoubiquitinated SPRTN in cells and *in vitro*. Depletion of USP11 increased SPRTN auto-proteolysis and sensitized cells to DPC-inducing agents leading to increased accumulation of DPCs. DPCs, if left unrepaired, lead to DNA breaks leading to genome instability. Consistent with this, it has been shown that USP11 depletion results in increased chromosomal abnormalities upon treatment with CPT and VP16 (42). Thus, in the absence of USP11, SPRTN is inactivated by increased auto-proteolysis, which may lead to inefficient SPRTN-mediated DPC repair, generating genomic instability leading to cancer and premature aging.

SPRTN-mediated DPC repair and regulation of SPRTN protease are only beginning to be understood. Given that SPRTN is a sequence nonspecific metalloprotease, promotes the repair of enzymatic and nonenzymatic DPCs, and participates in proteasome-dependent and -independent DPC repair processes, a tight regulation of SPRTN recruitment and activity are critical to prevent aberrant SPRTN protease activity on chromatin-bound proteins. SPRTN is constitutively monoubiquitinated (34), and this ubiquitin switch is critical for SPRTN function. Therefore, multiple DUBs that deubiquitinate SPRTN, namely VCPIP1 (35), USP7 (34), and USP11 (this study), have been identified.

Recently, it was shown that USP7 deubiquitinates SPRTN, preventing SPRTN auto-cleavage upon DPC induction. We observed that USP11 depletion also promotes SPRTN auto-cleavage, and auto-proteolysis of SPRTN is exacerbated in the combined absence of USP11 and USP7. USP11 and USP7 interact and have common targets such as XPC and components of the PRC1 complex. Further, both USP11 and USP7 regulate PML, though with opposite effects (49, 50, 51). Our study adds monoubiquitinated SPRTN to the list of shared targets of USP11 and USP7. Zhao *et al.* (34) showed that unmodified SPRTN does not undergo auto-cleavage and that monoubiquitination inactivates SPRTN by promoting both auto-proteolysis and polyubiquitination, which targets SPRTN for degradation. Thus, deubiquitination of SPRTN upon DPC induction prevents SPRTN inactivation. In undamaged conditions, it is speculated that monoubiquitin on SPRTN is shielded from polyubiquitination as a consequence of being bound by the UBZ domain of SPRTN. However, in undamaged conditions, we observe increased SPRTN auto-proteolysis in the absence of USP11 or USP7, which is further increased upon depletion of both USP11 and USP7. Moreover, we observe SPRTN interaction with USP11 in the absence and upon DNA damage. This brings about a possibility that USP11 and USP7 may be required to regulate SPRTN auto-proteolysis even in the absence of damage and that deubiquitination of SPRTN may not be restricted to signaling from DPC induction alone. We observed reduced SPRTN auto-cleavage upon DPC induction in USP11 and USP7 single and double-knockdown cells, suggesting that in the absence of USP11 and USP7, SPRTN could be deubiquitinated by VCPIP1, which is recruited to chromatin upon DPC induction.

Previously, it was proposed that monoubiquitination prevents SPRTN recruitment to chromatin (4). However, we observe that lack of SPRTN deubiquitination by USP11 and VCPIP1 did not affect recruitment on chromatin. Similarly, Zhao *et al.* (34) showed that deubiquitination of SPRTN by USP7 does not govern SPRTN localization on chromatin. These studies suggest that monoubiquitination does not restrict SPRTN access to chromatin. Huang *et al.* (35) showed that SPRTN chromatin recruitment is regulated by two-step posttranslational modification, deubiquitination followed by acetylation. A previous study showed that SPRTN associates with the replisome and localizes on chromatin in the absence of damage (18). Further, Halder *et al.* (33) showed that monoubiquitinated and unmodified SPRTN is localized on chromatin in the absence of damage, wherein during DNA replication, SPRTN cleaves the C-terminal-inhibitory part of CHK1, releasing CHK1 from replicative chromatin. In a feedback mechanism, CHK1 phosphorylates SPRTN at the C terminus, stimulating SPRTN recruitment to chromatin to promote unperturbed DNA replication fork progression and DPC repair. All these observations suggest that either monoubiquitinated SPRTN localized on chromatin in the absence of damage is deubiquitinated upon DPC induction, enriching the unmodified SPRTN on chromatin and acetylation of SPRTN retains SPRTN on chromatin, or SPRTN deubiquitination and acetylation occur in parallel to SPRTN recruitment. Further, phosphorylation, but not deubiquitination of SPRTN, could serve as the signal for SPRTN recruitment to chromatin in the absence and upon DNA damage (33). However, these possibilities need to be extensively investigated.

**Figure 7:**
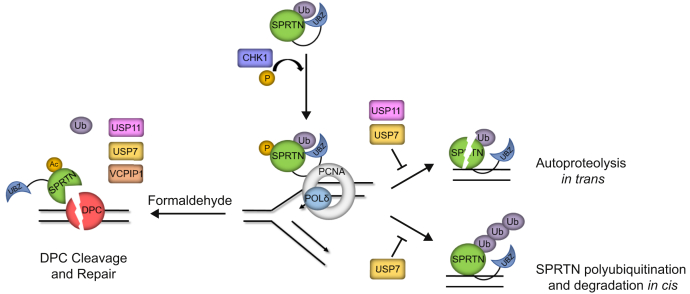
**Model of SPRTN regulation by deubiquitinases**.

Based on our study and the observations made in previous studies, we propose a unified model for SPRTN regulation by DUBs (Fig. 7). SPRTN is constitutively monoubiquitinated (34). During normal DNA replication, SPRTN associates with the replisome on chromatin to regulate DNA fork progression and DPC repair. CHK1 phosphorylates the C terminus of SPRTN and aids SPRTN recruitment to chromatin (33). The monoubiquitination of SPRTN promotes SPRTN inactivation by promoting SPRTN auto-proteolysis *in trans* (34). The negative regulation is relieved by deubiquitination of SPRTN by USP11 (this study) and USP7 (34). Monoubiquitin on SPRTN also promotes polyubiquitination targeting SPRTN for degradation in *cis*, which is counteracted by SPRTN deubiquitination by USP7. It is speculated that monoubiquitin on SPRTN is shielded by the UBZ domain of SPRTN (23, 34). Upon DPC induction, UBZ domain of SPRTN binds other ubiquitinated substrates, uncovering the monoubiquitin to be removed by deubiquitinase(s). Upon formaldehyde treatment and other yet to be identified signals, SPRTN is deubiquitinated by USP11 (this study), USP7, and VCPIP1 (34, 35). Deubiquitination of SPRTN is a prerequisite to SPRTN acetylation by PCAF and GCN5 recruited to SPRTN by VCPIP1 (35). Unmodified SPRTN enriched on the chromatin upon DPC-induction proteolyzes proteins cross-linked to DNA to repair DPCs.

Identification of multiple DUBs and posttranslational modifications of SPRTN have opened up the field to several unanswered questions on SPRTN regulation and DPC repair. Future studies will involve the identification of DPC sensors/effectors, deubiquitinases activated by specific types of DPC-lesions, and an in-depth analysis on the effect of posttranslational modifications (ubiquitination, sumoylation, acetylation, and phosphorylation) of SPRTN on SPRTN activity and function. Multiple DUBs regulate SPRTN, but the cross talk and regulation of these DUBs need to be investigated. The activation and preference of these DUBs to function in response to all or select types of DPCs remain unknown. Further, the identification of the E3 ligase for SPRTN will be critical in deciphering the dynamic regulation of SPRTN ubiquitination and function in DPC repair. The cross talk between ubiquitination–deubiquitination, acetylation, and phosphorylation on SPRTN protease activity, auto-proteolysis, and recruitment to DPC lesions remains to be examined.

## Experimental procedures

### Cell culture and transfections

HEK 293T, U2OS, and A549 cell lines (ATCC; Cat#CRL-11268, Cat#HTB-96, Cat#CCL-185) were cultured in Dulbecco’s Modified Eagle medium (DMEM, Hyclone) supplemented with 10% fetal bovine serum (FBS, Hyclone) and 1% penicillin/streptomycin (Gibco). HCT116 cell line (ATCC; Cat#CCL-247) was cultured in McCoy’s 5A supplemented with 10% FBS and 1% penicillin/streptomycin. Cells were maintained in a 37 °C incubator containing 5% CO_2_. Transfection of expression vectors was carried out using Lipofectamine 2000 (Invitrogen) or PEI (Polysciences, Inc) reagents as per the protocol from the manufacturer. Non-targeting control siRNA and siRNA targeting USP11 were transfected into cells using Lipofectamine RNAi MAX (Invitrogen) following the manufacturer’s protocol. See Table S4 for a complete list of siRNA targeting sequences used in this study.

### Plasmid cloning and site-directed mutagenesis

All cDNAs were subcloned into pDONR201 vector as entry clones and N-terminal tagged fusion constructs were generated by transferring the gene insert from the entry clones into gateway-compatible destination vectors by gateway cloning strategy using BP clonase and LR clonase enzymes (Thermo Fisher Scientific; Cat#11789021, Cat#11791100) as per the manufacturer’s instructions. All deletion and point mutants were generated by Quick change site-directed mutagenesis protocol using KOD Hot Start Polymerase (Sigma; Cat#71086) and DpnI (New England Biolabs; Cat#R0176 L) digestion. All constructs generated in this study were confirmed by DNA sequencing. See Table S4 for a complete list of primers used for site-directed mutagenesis.

### Stable cell line generation

Stable shRNA knockdown cell lines were generated by lentiviral transduction of shRNA targeted to USP11 or nonsilencing control. Virus was produced in HEK 293T cells transfected with the shRNA plasmid, psPAX2 and pMD2.G lentiviral packaging plasmids in a ratio of 4:2:1. Forty-eight hours posttransfection, virus was collected and used to transduce U2OS, A549, HEK 293T, or HCT116 cells. GFP-positive cells were sorted by flow cytometry into 96-well plates, and stable clones were selected with puromycin-containing media. See Table S4 for shRNA sequences. Stable overexpression cell lines were generated by transfecting HEK 293T with SFB-tagged constructs and seeding cells at low density in puromycin-containing media 24 h posttransfection. Individual clones were isolated and screened for expression. Puromycin (Sigma-Aldrich; Cat#P7255) concentrations used for stable cell lines are as follows: U2OS, 1.2 μg/ml; A549, 3.0 μg/ml; HEK 293T, 1.0 μg/ml; HCT116 0.25 μg/ml.

### Tandem affinity purification–mass spectrometry

TAP was performed as described previously (21). HEK 293T cells were transfected with plasmids encoding SFB (S-protein, FLAG, and streptavidin-binding peptide)-tagged constructs. Cell lines stably expressing tagged proteins were selected, and the expression of exogenous proteins was confirmed by immunoblotting and immunostaining. For affinity purification, HEK 293T cells stably expressing SFB-tagged protein from a total of 20 10-cm dishes were collected and lysed in NETN buffer (250 mM NaCl, 5 mM EDTA pH 8.0, 50 mM Tris-HCl pH 8.0, and 0.5% Nonidet P-40) supplemented with protease inhibitor cocktail (Roche). Crude lysates were cleared by centrifugation, and the supernatants were incubated with 200 μl of streptavidin-Sepharose beads (Sigma-Aldrich; Cat#GE17-5113-01) overnight at 4 °C. The beads were washed three times with NETN buffer and then eluted with 2 mg/ml biotin (Sigma-Aldrich; Cat#B4501) for 2 h at 4 °C. The eluates were incubated with 40 μl of S-protein-agarose beads (Millipore; Cat#69704) for 2 h at 4 °C and then washed three times with NETN buffer. The proteins bound to beads were eluted by boiling with 4X Laemmli buffer (SDS sample buffer), resolved by SDS-PAGE, and visualized by Coomassie Blue staining. Excised gel bands were cut into approximately 1 mm^3^ pieces. Gel pieces were then subjected to a modified in-gel trypsin digestion procedure. Gel pieces were washed and dehydrated with acetonitrile for 10 min followed by removal of acetonitrile. Pieces were then completely dried in a speed-vac. Rehydration of the gel pieces was with 50 mM ammonium bicarbonate solution containing 12.5 ng/μl modified sequencing-grade trypsin (Promega) at 4 °C. After 45 min, the excess trypsin solution was removed and replaced with 50 mM ammonium bicarbonate solution to just cover the gel pieces. Samples were then placed in a 37 °C room overnight. Peptides were later extracted by removing the ammonium bicarbonate solution, followed by one wash with a solution containing 50% acetonitrile and 1% formic acid. The extracts were then dried in a speed-vac (∼1 h). The samples were then stored at 4 °C until analysis.

On the day of analysis, the samples were reconstituted in 5 to 10 μl of HPLC solvent A (2.5% acetonitrile, 0.1% formic acid). A nanoscale reverse-phase HPLC capillary column was created by packing 2.6 μm C18 spherical silica beads into a fused silica capillary (100 μm inner diameter × ∼30 cm length) with a flame-drawn tip. After equilibrating the column, each sample was loaded *via* a Famos autosampler (LC Packings) onto the column. A gradient was formed and peptides were eluted with increasing concentrations of solvent B (97.5% acetonitrile, 0.1% formic acid).

As peptides eluted, they were subjected to electrospray ionization and then entered into an LTQ Orbitrap Velos Pro ion-trap mass spectrometer (Thermo Fisher Scientific). Peptides were detected, isolated, and fragmented to produce a tandem mass spectrum of specific fragment ions for each peptide (Peaklist-generating software: ReAdW.exe, version 4.3.1) Peptide sequences (and hence protein identity) were determined by matching protein databases with the acquired fragmentation pattern by the software program, SEQUEST (Thermo Fisher Scientific; ver. 28, rev 13). The searched database was downloaded from Uniprot on June 20, 2017, which contained 160,020 entries with 138 entries added as a contaminant list. All databases include a reversed version of all the sequences. The maximum number of missed and/or nonspecific cleavages permitted was set at 2. The only fixed modification considered was Cys −57.0214 Da (iodoacetamide) and variable modification considered was Met −15.9949 Da (oxidation). Mass tolerance for precursor ions was 50 ppm and for fragments ions was 1 Da. The cutoff threshold for accepting individual spectra was a mass accuracy of below 10 ppm of the expected along with a minimum xCorr of 1.0 for +2, and 2.0 for +3 and +4 charge state peptides. The SFB-SPRTN estimation of false discovery rate is 0.37% and 0.25% for SFB-USP11, calculated by the (number of reverse peptides divided by total peptides)∗2 (52, 53, 54).

### Immunoblotting and antibodies

Cells were lysed with NETN buffer on an end-to-end rocker at 4 °C for 30 min. Cleared cell lysates were then collected by centrifugation, boiled in SDS sample buffer, and separated by SDS-PAGE. Proteins were transferred to PVDF membrane (Millipore) *via* semidry transfer. Membranes were blocked in 5% milk in 1X TBS/Tween buffer (TBST) and probed with antibodies as indicated in the figures.

The following primary antibodies were used in this study: anti-FLAG (Sigma-Aldrich F3165; dilution 1:10,000); anti-c-Myc (Santa Cruz Biotechnology sc-40; dilution 1:1000); anti-β-Actin (Sigma-Aldrich A5441; dilution 1:10,000); anti-α-Tubulin (Sigma-Aldrich T6074; dilution 1:5000); anti-HA-Tag (Cell Signaling Technology 3724S; dilution 1:2000); anti-Ubiquitin (FK2) (Millipore ST1200; dilution 1:1000); anti-K63-linkage Specific Polyubiquitin (Cell Signaling Technology 5621S; dilution 1:1000); anti-USP11 (Bethyl Laboratories A301-613A; dilution 1:1000); anti-SPRTN (Invitrogen PA5-46262; dilution 1:1000); anti-Topoisomerase I (Abcam ab109374; dilution 1:2000); anti-Histone H3 (Cell Signaling Technology 9715S; dilution 1:2000); anti-VCIP135 (Cell Signaling Technology 88153S; dilution 1:1000); anti-USP7 (Bethyl A300-033A dilution 1:7500); anti-Topoisomerase II-alpha (Cell Signaling Technology 12286S; dilution 1:1000); anti-dsDNA (Abcam ab27156; dilution 1:10,000). Secondary antibodies used in this study are as follows: Peroxidase-conjugated AffiniPure Polyclonal Goat anti-Rabbit IgG (Jackson ImmunoResearch Laboratories 111-035-144; dilution 1:10,000); Peroxidase-Conjugated AffiniPure Polyclonal Rabbit anti-Mouse IgG + IgM (Jackson ImmunoResearch Laboratories 315-035-048; dilution 1:10,000).

### Co-immunoprecipitations

HEK 293T cells were transfected with constructs encoding SFB and Myc-tagged proteins and incubated for 24 h. Cells were lysed with NETN buffer. The lysates were cleared by centrifugation and incubated with 20 μl of S-protein agarose beads overnight on an end-to-end rocker at 4°C. After three washes with NETN buffer, the proteins bound to beads were eluted by boiling with SDS sample buffer, resolved by SDS-PAGE, and analyzed by western blotting.

### Clonogenic cell survival assays

In total, 800 cells were seeded in 60 mm dishes in triplicate. Twenty-four hours after seeding, cells were treated with CPT (Sigma-Aldrich; Cat#C9911), VP16 (Sigma-Aldrich; Cat#E1383), formaldehyde (Sigma-Aldrich; Cat#F8775), HU (Sigma-Aldrich; Cat#H8627), or MMC (Sigma-Aldrich, Cat#M4287) at the indicated concentrations and for the indicated times. Cells were washed with PBS, supplemented with fresh media, and incubated for 10 to 14 days. Formed colonies were fixed and stained with Coomassie Blue. The numbers of colonies were counted, and the percentage cell survival was calculated.

### *In vitro* deubiquitination assay

The *in vitro* deubiquitination was performed as described (55). SFB-SPRTN alone or HA-Ubiquitin and SFB-SPRTN together were expressed in HEK 293T cells. At 24 h posttransfection, cells were lysed in NETN buffer. SFB-tagged ubiquitinated SPRTN was purified by immunoprecipitation with streptavidin-sepharose beads followed by elution with 2 mM biotin on an end-to-end rocker at 4 °C for 1 h. The eluate for SFB-HA-Ub-SPRTN was then immunoprecipitated with anti-HA-beads (Thermo Fisher Scientific; Cat#88836) and eluted with 2 mg/ml HA peptide (Sigma-Aldrich; Cat#I2149) at 37 °C for 10 min. In a parallel experiment, Myc-USP11 and Myc-USP11 C318S were expressed in HEK 293T cells for 24 h. Myc-USP11 or Myc-USP11 C318S was purified by immunoprecipitation with anti-Myc-agarose beads (Thermo Fisher Scientific; Cat#20168) followed by elution with 100 μg/ml c-Myc peptide (Sigma-Aldrich; Cat#M2435) on an end-to-end rocker at room temperature for 1 h. For *in vitro* deubiquitination assay, purified SFB-SPRTN or SFB-HA-Ub-SPRTN was incubated with purified Myc-USP11 or Myc-USP11 C318S in a deubiquitination reaction buffer (50 mM HEPES, pH 7.5, 100 mM NaCl, 5% glycerol, 5 mM MgCl_2_, 1 mM ATP, and 1 mM DTT) at 30 °C overnight. The reaction mixture was terminated using SDS-PAGE buffer and analyzed by western blotting.

### Deubiquitination assay in cells

HEK 293T cells were transfected with constructs encoding SFB-SPRTN, Myc-tagged USP11 FL or C318S, and HA-Ubiquitin, where indicated. Cells were lysed 24 h later with NETN buffer. The lysates were cleared by centrifugation and then incubated with 20 μl of S-protein agarose beads on an end-to-end rocker overnight at 4 °C. After three washes with NETN buffer, the proteins bound to beads were eluted by boiling with SDS sample buffer, resolved by SDS-PAGE, and analyzed by western blotting.

### Soluble and chromatin fractionation

Preparation of cellular fractions was performed as previously described with modifications (56). For total cell lysate, cell pellets were directly lysed in three volumes of NETN and one volume of SDS sample buffer. For soluble and chromatin fractions, cell pellets were resuspended in four volumes of low salt fractionation buffer (10 mM Tris-HCl pH 7.4, 0.2 mM MgCl_2_, 1% Triton-X 100, and protease inhibitors) and incubated at 4 °C for 20 min. The chromatin fraction was pelleted by centrifugation (13,000 rpm for 10 min), and the supernatant was collected as the soluble fraction. The chromatin pellet was resuspended in four volumes of 0.2 N HCl and incubated on ice for 20 min with intermittent vortexing. The samples were centrifuged (13,000 rpm for 10 min), the supernatant was collected as the chromatin fraction and neutralized with an equal volume of 1 M Tris-HCl pH 8.0.

### RADAR assay and slot blotting

RADAR protocol was performed as described (57). 1.5 × 10^5^ cells were seeded in 6-well plates, grown to 80% confluence and then treated with CPT or VP16 at the indicated concentrations and time. The cells were directly lysed in 0.5 ml of DNAzol (Invitrogen; Cat#10503027). Genomic DNA and DNA–protein covalent complexes were precipitated from the lysate by addition of 0.5 volume of 100% ethanol followed by a 10 min incubation at −20 °C. The precipitate was collected by centrifugation at 7000 rpm for 5 min, washed twice in 70% ethanol, and immediately resuspended in 300 μl of freshly prepared 8 mM NaOH. The DNA content of each sample was measured with a NanoDrop One instrument (ThermoFisher Scientific) or PicoGreen dsDNA Assay Kit (ThermoFisher Scientific; Cat #P7589). Samples were diluted in 25 mM sodium phosphate buffer pH 6.5 to a final volume of 200 μl. Sample was applied to nitrocellulose membrane (Amersham) using a vacuum slot-blot manifold (Hoefer PR648). Anti-dsDNA loading control samples were applied to positively charged nylon membrane (Invitrogen; Cat#AM10104). The membrane was blocked in 5% milk in 1X TBST and incubated with antibodies as described for immunoblotting. TOP1-cc or TOP2-cc fold change was quantified from relative abundance of TOP1 or TOP2 and normalized to the loading control or to the amount of DNA loaded.

### ARK assay

ARK assay was performed as described (48). U2OS (5 × 10^5^) cells lysed in 700 μl of prewarmed M Buffer (5.6 M GTC, 10 mM Tris-HCl (pH 6.5), 20 mM EDTA, 4% Triton X-100, 1% Sarkosyl, and 0.1% dithiothreitol) were collected after scraping and mild shearing with a 1-ml pipet tip. The lysates were sheared by passing through a 22-gauge needle six times followed by DNA precipitation with an equal volume of prechilled ethanol (−20 °C). Free DNA and DPCs were recovered as a pellet after centrifugation at 12,000 rpm, 4 °C for 20 min. The pellet was washed in the wash buffer (20 mM Tris–HCl pH 6.5, 150 mM NaCl, and 50% ethanol). The pellet was dissolved in 0.5 μl of prewarmed Buffer A composed of 1% SDS and 20 mM Tris-HCl (pH 7.5) and incubated at 42 °C for 6 min. The samples passed through a 25-gauge needle 5 times and SDS-bound DPCs were precipitated with 0.5 μl of Buffer B (200 mM KCl and 20 mM Tris-HCl (pH 7.5)) on ice for 6 min, followed by centrifugation at 12,000 rpm for 5 min at 4 °C. The supernatant was collected and the DPC pellet was washed twice in Buffer C (1.5 ml of 100 mM KCl and 20 mM Tris-HCl (pH 7.5)) at 55 °C for 10 min, on ice for 6 min, followed by centrifugation at 12,000 rpm at 4 °C for 10 min. The supernatant was collected and combined with the previously collected supernatant for total DNA measurement. DPC pellet was dissolved in 1 ml of Buffer D (100 mM KCl, 20 mM Tris-HCl, pH 7.5, and 10 mM EDTA). Proteinase K was added to a final concentration of 0.2 mg/ml and incubated at 55 °C for 45 min. Samples were chilled on ice for 6 min and centrifuged at 12,000 rpm for 10 min at 4 °C. The supernatant containing DPC-associated DNA was collected and the DPC coefficient was determined by DNA quantification by the PicoGreen assay kit. Ten microliters from the 4 ml of the recovered free DNA supernatant and 63 μl from the 1 ml supernatant of the DPC resuspension were used for DNA quantification. The DPC coefficient is expressed as the percentage of DNA from DPCs over the total DNA in each sample.

### Quantification and statistical analysis

All experiments were independently replicated (biological replicates) at least three times. Technical replicates for clonogenic cell survival assays and RADAR assays are indicated in the figure legends. Western blots and slot blots were quantified using ImageJ software and normalized to the loading control or as indicated in the figure legends. Results were graphed and analyzed using GraphPad Prism 8 software and data are presented as mean ± SD. Two-tailed paired *t*-test was performed for clonogenic cell survival assays, and two-way ANOVA and Dunnett’s multiple comparison test were performed for RADAR and ARK assays. All data points were included in determining the *p*-values with a confidence interval set to 90% and α = 0.1 to account for any variabilities due to data point outliers. Statistical significance was reported as *p* ≤ 0.1(∗), *p* ≤ 0.05 (∗∗), *p* ≤ 0.01 (∗∗∗), *p* ≤ 0.001 (∗∗∗∗) and n.s. as not statistically significant.

## Data availability

The mass spectrometry proteomics data have been deposited to the ProteomeXchange Consortium *via* the PRIDE (58) partner repository with the data set identifier PXD019923. Further information and requests of materials used in this research should be directed to Gargi Ghosal (gargi.ghosal@unmc.edu). Plasmid DNA constructs generated in this study (indicated in Table S4) will be made available *via* material transfer agreement (MTA).

## Conflict of interest

The authors declare that they have no conflicts of interest with the contents of this article.
