# Supplementary material for: USP11 mediates repair of DNA–protein cross-links by deubiquitinating SPRTN metalloprotease
Source: J Biol Chem. 2021 Feb 7;296:100396. doi: 10.1016/j.jbc.2021.100396 (PMC7960550; doi:10.1016/j.jbc.2021.100396)
Supplement: Supplemental Figures S1–S8 and Tables S1–S4 [file mmc1.pdf]

Supporting information for:

USP11 deubiquitinates monoubiquitinated SPRTN to repair DNA-protein crosslinks

Megan Perry[1], Meghan Biegert[1], Sai Sundeep Kollala[1], Halle Mallard[1], Grace Su[2], Manohar Kodavati[3], Natasha Kreiling[1], Alexander Holbrook[1], and Gargi Ghosal[1,4*]

[1]Department of Genetics, Cell Biology and Anatomy, University of Nebraska Medical Center, Omaha, NE 68198

[2]Department of Biology, Doane University, Crete, NE 68333

[3]Department of Radiation Oncology, Houston Methodist Research Institute, Houston, TX 77030

[4]Fred and Pamela Buffett Cancer Center, Omaha NE 68105

[*]Corresponding author: Gargi Ghosal

E-mail: gargi.ghosal@unmc.edu

Content:

- Supporting information figures S1-S8
- Supporting information Tables S1-S4

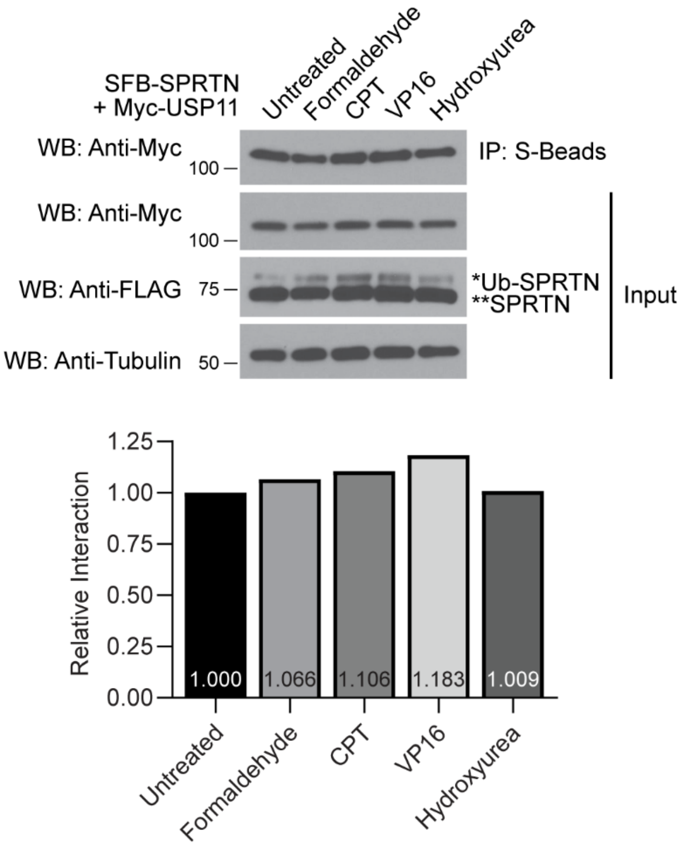

Figure S1 – related to Figure 1. SPRTN-USP11 interaction in the presence of DNA damaging agents.

(A) HEK 293T cells were transfected with SFB-SPRTN and Myc-USP11. Cells were treated with 3 mM formaldehyde, 10 μM CPT, 10 μM VP16 or 2 mM hydroxyurea for 1 hr. Top panel, cell lysates were immunoprecipitated with S-protein agarose beads and immunoblotted with indicated antibodies. Bottom panel, quantification of interaction of Myc USP11 and SFB SPRTN relative to the untreated sample and normalized to Myc input WB. WB, Western Blot.

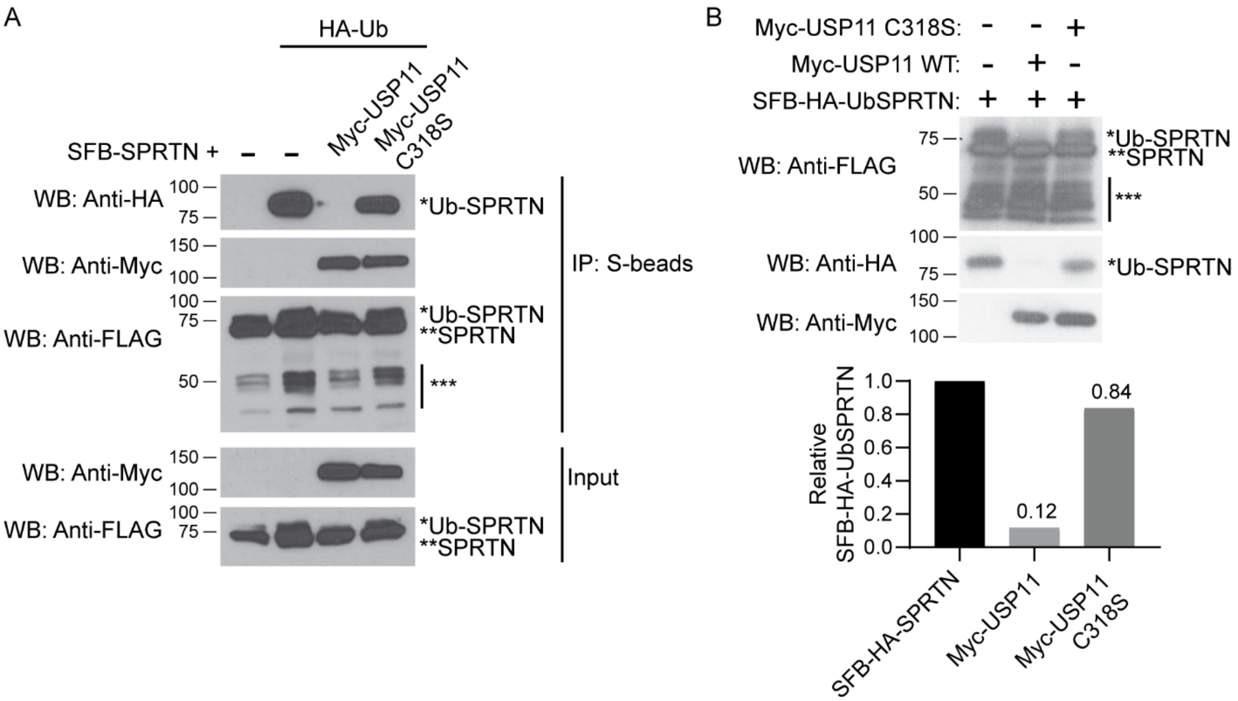

Figure S2 – related to Figure 3. USP11 deubiquitinates SFB-HA-UbSPRTN.

(A) HEK 293T cells were transfected with SFB-SPRTN, HA-Ubiquitin and Myc-USP11 FL or C318S. Cell lysates were immunoprecipitated with S-protein agarose beads and immunoblotted with indicated antibodies. (B) Top panel, SFB-HA-UbSPRTN, Myc-USP11, and Myc-USP11 C318S were purified as described in methods. SFB-HA-UbSPRTN was incubated with Myc-USP11 or Myc-USP11 C318S mutant proteins overnight at 30 °C. The reaction mixture was immunoblotted with indicated antibodies. Bottom panel, quantification of anti-HA WB, values relative to SFB-SPRTN alone were calculated and plotted as Relative SFB-HA-UbSPRTN. WB, Western Blot. ***indicates SPRTN auto-cleavage products.

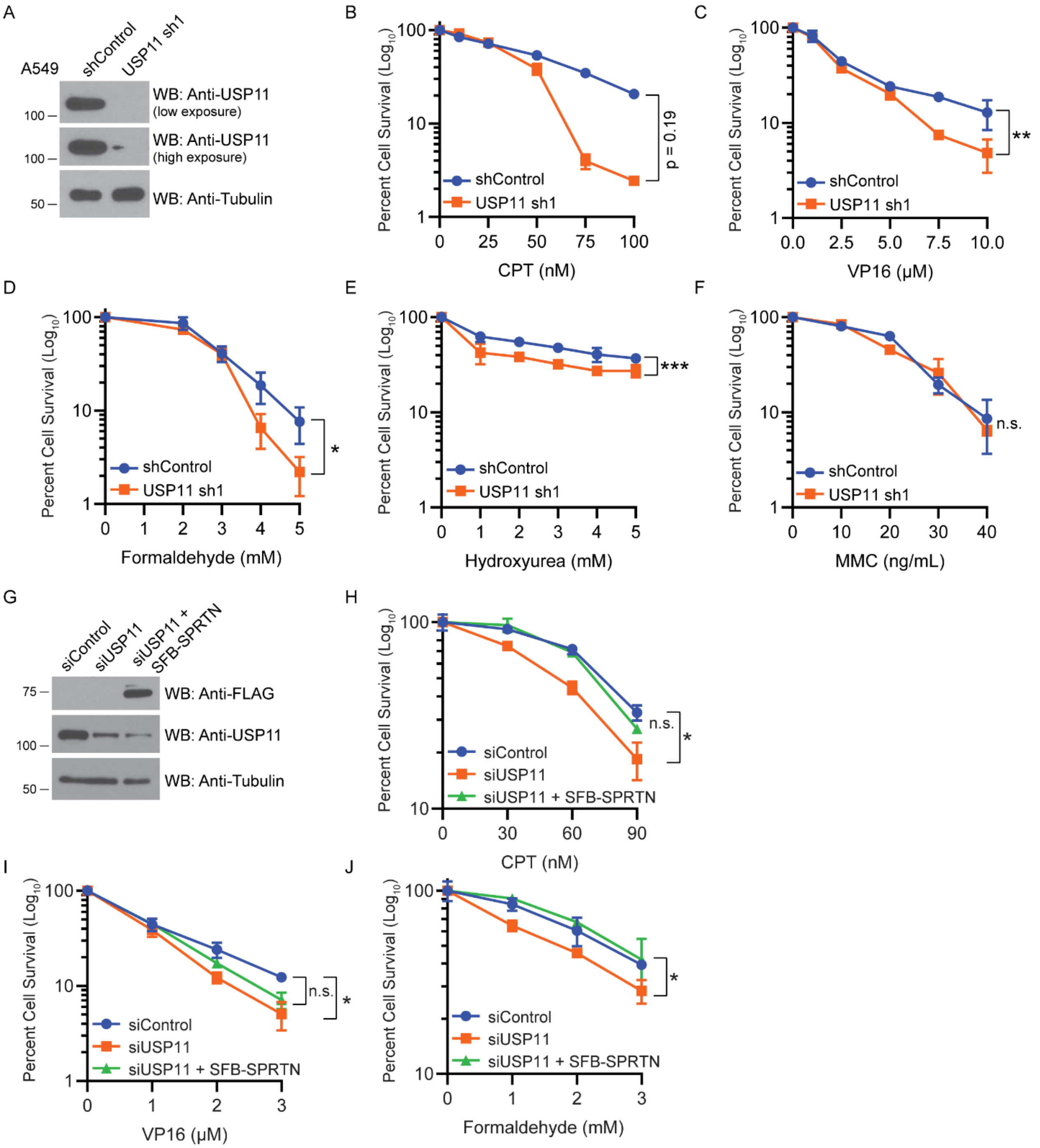
A
A549
shControl
USP11 sh1
WB: Anti-USP11
(low exposure)
WB: Anti-USP11
(high exposure)
WB: Anti-Tubulin
B
Percent Cell Survival (Log10)
shControl
USP11 sh1
p = 0.19
CPT (nM)
C
VP16 (μM)
**
D
Formaldehyde (mM)
*
E
Hydroxyurea (mM)
***
F
MMC (ng/mL)
n.s.
G
siControl
siUSP11
siUSP11 + SFB-SPRTN
WB: Anti-FLAG
WB: Anti-USP11
WB: Anti-Tubulin
H
CPT (nM)
n.s.
*
I
VP16 (μM)
J
Formaldehyde (mM)

Figure S3 – related to Figure 4. Depletion of USP11 sensitizes cells to treatment with DPC-inducing agents.

(A) WB showing knockdown efficiency of USP11 in A549 cells stably expressing non-silencing shRNA control or shRNA targeted to USP11. (B-F) Clonogenic cell survival curves for shRNA control and USP11 knockdown cells treated with indicated concentrations of (B) CPT for 24 hr, (C) VP16 for 4 hr, (D) formaldehyde for 20 min, (E) HU for 24 hr, or (F) MMC for 24 hr. (G) WB showing knockdown efficiency of USP11 in HEK 293T cells transfected with siRNA targeted to USP11 and overexpression of SFB-SPRTN. (H-J) Clonogenic cell survival curves for siRNA control, USP11 knockdown cells, and USP11 knockdown cells transfected with SFB-SPRTN treated with indicated concentrations of (H) CPT for 24 hr, (I) VP16 for 4 hr, or (J) formaldehyde for 20 min. (B-F, H-J) Percent cell survival was calculated and data are presented as mean ± SD ($n = 3$). Statistical analysis: Two-tailed paired t-test was performed using confidence interval = 90% and $\alpha=0.1$; *$p \leq 0.1$, **$p \leq 0.05$, ***$p \leq 0.01$; n.s., not statistically significant. WB, Western Blot.

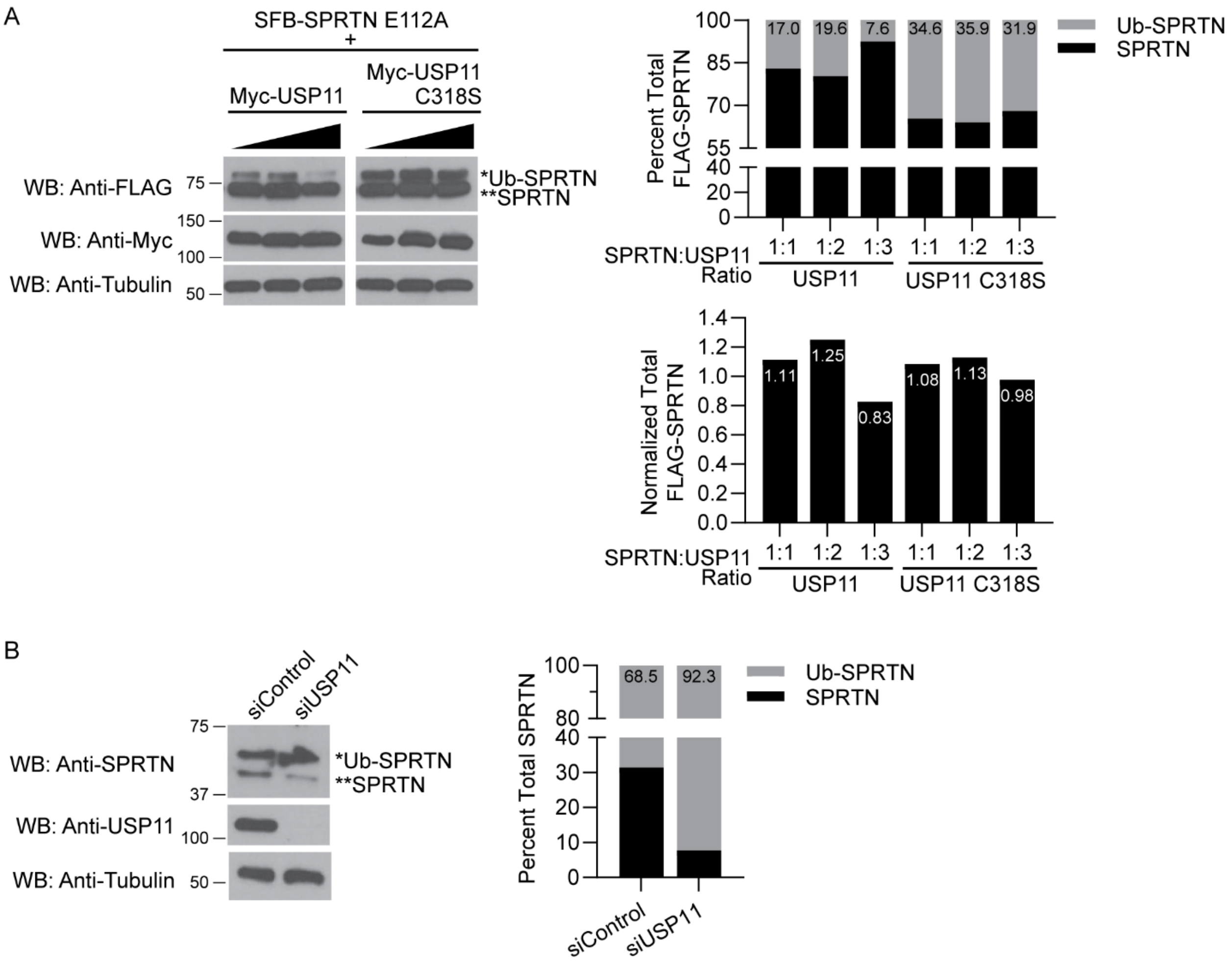
A
SFB-SPRTN E112A
+
Myc-USP11
Myc-USP11 C318S
WB: Anti-FLAG
WB: Anti-Myc
WB: Anti-Tubulin
75
150
100
50
*Ub-SPRTN
**SPRTN
Percent Total FLAG-SPRTN
100
85
70
55
40
20
0
17.0
19.6
7.6
34.6
35.9
31.9
Ub-SPRTN
SPRTN
SPRTN:USP11 Ratio
1:1
1:2
1:3
1:1
1:2
1:3
USP11
USP11 C318S
Normalized Total FLAG-SPRTN
1.4
1.2
1.0
0.8
0.6
0.4
0.2
0.0
1.11
1.25
0.83
1.08
1.13
0.98
SPRTN:USP11 Ratio
1:1
1:2
1:3
1:1
1:2
1:3
USP11
USP11 C318S
B
siControl
siUSP11
WB: Anti-SPRTN
WB: Anti-USP11
WB: Anti-Tubulin
75
37
100
50
*Ub-SPRTN
**SPRTN
Percent Total SPRTN
100
80
40
30
20
10
0
68.5
92.3
Ub-SPRTN
SPRTN
siControl
siUSP11

Figure S4 – related to figure 5. USP11 does not regulate SPRTN protein stability.

(A) Left panel, HEK 293T cells were transfected with SFB-SPRTN E112A (2.5 µg) and increasing concentrations (2.5, 5.0, or 7.5 µg) of Myc-USP11 FL or C318S mutant. Cell lysates were immunoblotted with indicated antibodies. Right top panel, quantification of anti-FLAG blots. The percent of unmodified and monoubiquitinated SPRTN was calculated. Graph shows percent total FLAG-SPRTN. Right bottom panel, quantification of anti-FLAG blots. Values of total SFB-SPRTN relative to 1:1 SPRTN:USP11 FL or C318S lanes were calculated and normalized with anti-tubulin blots and plotted as Normalized Total FLAG-SPRTN. (B) Left panel, A549 cells were transfected with siRNA targeted to USP11. 72 hr later, cell lysates were immunoblotted with the indicated antibodies. Right panel, quantification of anti-SPRTN blot. The percent of unmodified and monoubiquitinated SPRTN was calculated and plotted as percent total SPRTN.

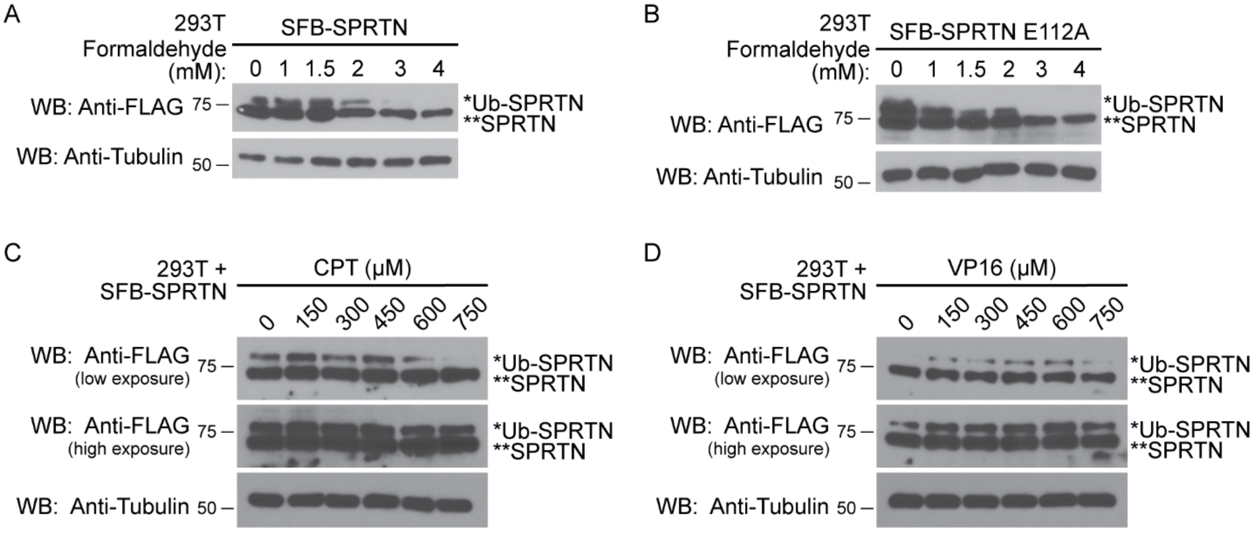

Figure S5 – related to Figure 5. SPRTN is deubiquitinated upon treatment with formaldehyde.

HEK 293T cells (A) transfected with SFB-SPRTN or (B) stably expressing SFB-SPRTN E112A were treated with the indicated concentrations of formaldehyde for 2 hr. (C-D) HEK 293T cells transfected with SFB-SPRTN were treated with the indicated concentrations of (C) CPT or (D) VP16 for 1 hr. (A-D) Cell lysates were immunoblotted with indicated antibodies. WB, Western Blot.

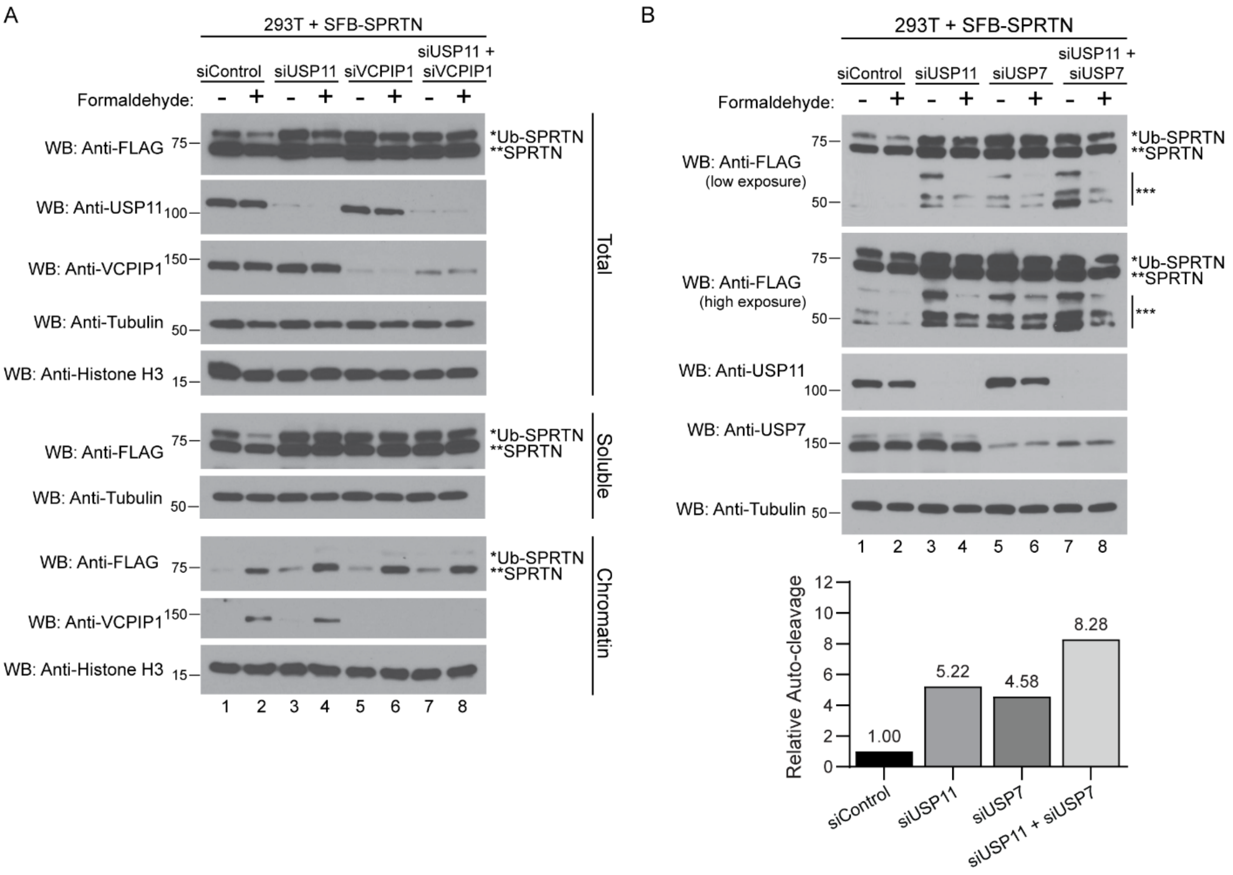

Figure S6 – related to Figure 5. USP11 regulates SPRTN auto-proteolysis.

(A) HEK 293T cells were transfected with siRNA targeted to USP11 and VCPIP1 alone or in combination. 48 hr later, cells were transfected with SFB-SPRTN. 72 hr post-siRNA transfection, cells were treated with 3 mM formaldehyde for 1 hr. Cells were fractionated into total, soluble and chromatin fractions. Fractions were immunoblotted with the indicated antibodies. (B) HEK 293T cells were transfected with siRNA targeted to USP11 and USP7 alone or in combination. 48 hr later, cells were transfected with SFB-SPRTN. 72 hr post-siRNA transfection, cells were treated with 3 mM formaldehyde for 1 hr. Top panel, the soluble fraction was isolated and immunoblotted with the indicated antibodies. Bottom panel, graph showing quantification of relative SPRTN autocleavage normalized to tubulin control in untreated samples (lanes 1, 3, 5, 7). Anti-FLAG high exposure was used for quantification. WB, Western Blot. ***indicates SPRTN auto-cleavage products.

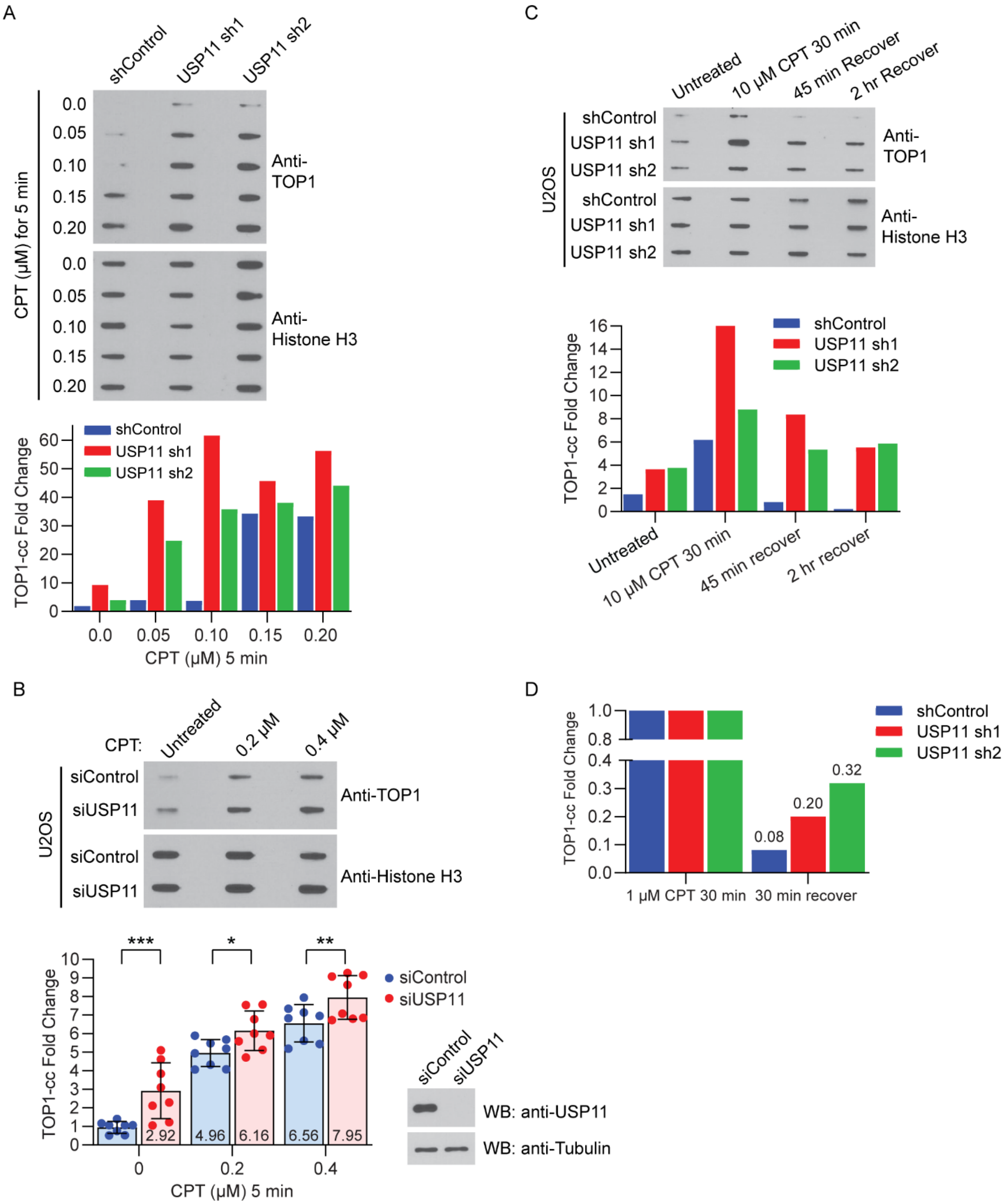
A
shControl
USP11 sh1
USP11 sh2
CPT (μM) for 5 min
0.0
0.05
0.10
0.15
0.20
Anti-TOP1
Anti-Histone H3
TOP1-cc Fold Change
CPT (μM) 5 min
B
CPT:
Untreated
0.2 μM
0.4 μM
U2OS
siControl
siUSP11
Anti-TOP1
Anti-Histone H3
***
*
**
2.92
4.96
6.16
6.56
7.95
WB: anti-USP11
WB: anti-Tubulin
C
Untreated
10 μM CPT 30 min
45 min Recover
2 hr Recover
Anti-TOP1
Anti-Histone H3
45 min recover
2 hr recover
D
1 μM CPT 30 min
30 min recover
0.08
0.20
0.32

Figure S7 – related to Figure 6. USP11 depleted cells accumulate TOP1-ccs.

(A-D) RADAR assay in U2OS cells (A, C, D) stably expressing non-silencing shRNA control or two different shRNA sequences (sh1 and sh2) targeted to USP11 or (B) transfected with siRNA targeted to USP11. Top panel, (A, B) cells were treated with CPT at the indicated concentrations for 5 min. 300 ng of DNA was immunoblotted with TOP1 antibody and 600 ng of DNA was immunoblotted with histone H3. (C-D) Cells were treated with 10 μM (C) or 1 μM (D) CPT for 30 min. Cells used for recovery time points were washed with PBS and left to recover in drug-free media for the indicated time. 300 ng of DNA was immunoblotted with TOP1 antibody and with histone H3. (A-D) Bottom panel, TOP1-cc fold change from slot blots shown above was quantified from relative abundance of TOP1 normalized to histone H3 (A, C, D) or normalized to amount of DNA loaded (B, n=8). Data in (B) are presented as mean ± SD. Statistical analysis: Two-way ANOVA and Dunnett's multiple comparison test was performed using confidence interval = 90% and $\alpha = 0.1$; $*p \leq 0.1$, $**p \leq 0.05$, $***p \leq 0.01$.

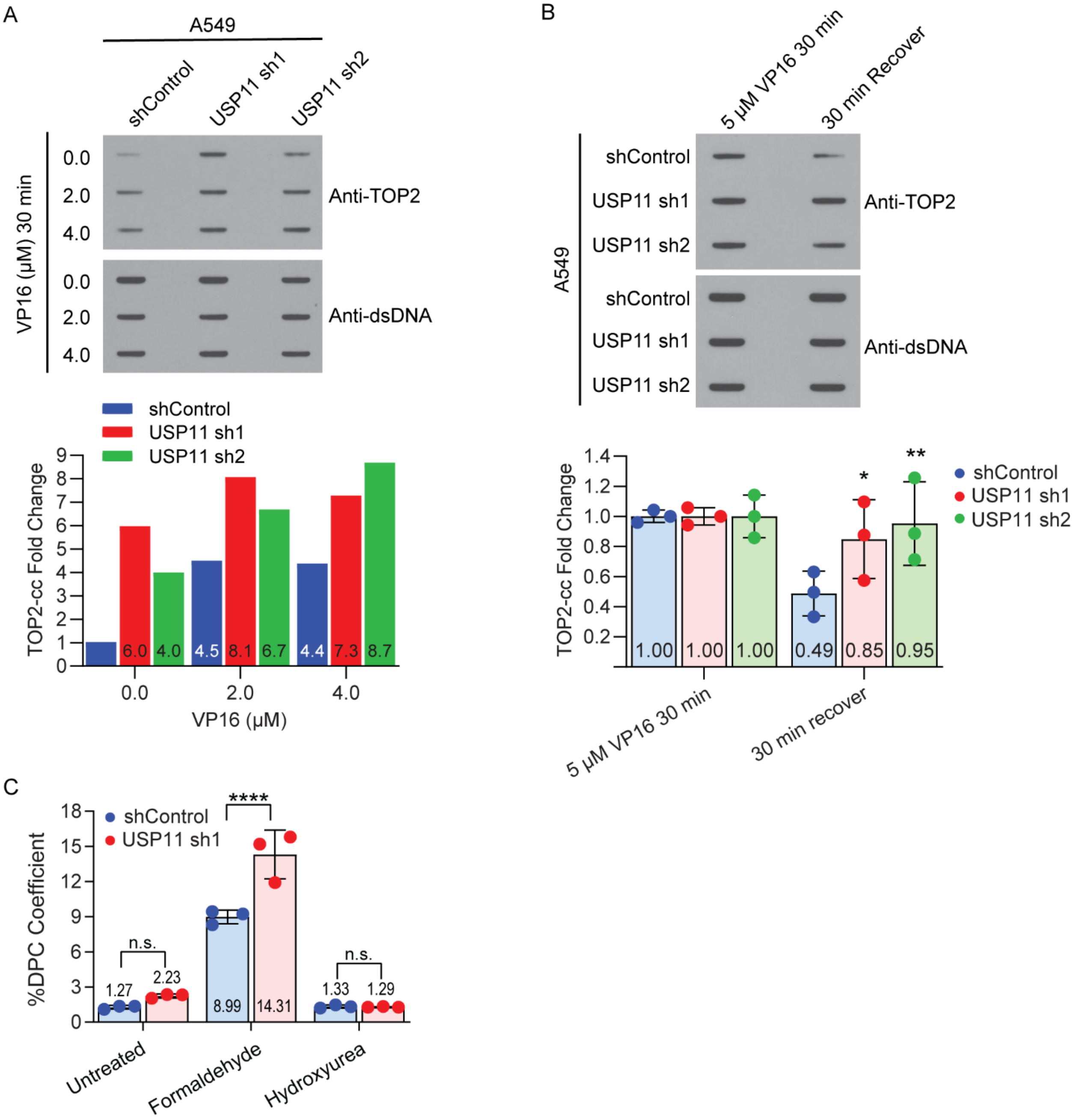
A
A549
shControl
USP11 sh1
USP11 sh2
VP16 (μM) 30 min
0.0
2.0
4.0
Anti-TOP2
Anti-dsDNA
TOP2-cc Fold Change
VP16 (μM)
6.0 4.0 4.5 8.1 6.7 4.4 7.3 8.7
B
5 μM VP16 30 min
30 min Recover
A549
30 min recover
1.00 1.00 1.00 0.49 0.85 0.95
*
**
C
%DPC Coefficient
n.s.
****
1.27
2.23
8.99
14.31
1.33
1.29
Untreated
Formaldehyde
Hydroxyurea

Figure S8 – related to Figure 6. USP11 depleted cells accumulate TOP2-ccs and non-specific DPCs.

(A-B) RADAR assay in A549 cells stably expressing non-silencing shRNA control or two different shRNA sequences (sh1 and sh2) targeted to USP11. Top panel, (A, B) cells were treated with VP16 at the indicated concentrations for 30 min. Cells used for recovery time points were washed with PBS and left to recover in drug-free media for the indicated time. 300 ng of DNA was immunoblotted with TOP2 antibody and 100 ng of DNA was immunoblotted with dsDNA antibody. Bottom panel, (A-B) TOP2-cc fold change from slot blots shown above was quantified from relative abundance of TOP2 normalized to dsDNA. (C) ARK assay performed in A549 USP11 stable knockdown cells treated with formaldeyhyde or hydroxyurea (2 mM for 2 hr). Data (B, n = 3; and C, n = 3) are presented as mean ± SD. Statistical analysis: Two-way ANOVA and Dunnett's multiple comparison test was performed using confidence interval = 90% and $\alpha = 0.1$; *$p \leq 0.1$, **$p \leq 0.05$, ****$p \leq 0.001$.

Table S1: SPRTN MS analysis results (.xlsx file)

Table S2: Annotated, mass-labeled MS/MS spectra for POLD3 and USP11 (.pdf file)

Table S3: USP11 MS analysis results (.xlsx file)

Table S4: Recombinant DNA, Oligonucleotides and Primers

**TABLE S4**		
**Recombinant DNA**	**SOURCE**	**IDENTIFIER**
Vector: SFB Destination	Dr. Junjie Chen	(Ghosal et al., 2012)
Vector: Myc Destination	Dr. Junjie Chen	(Ghosal et al., 2012)
Vector: pDNR201	Dr. Junjie Chen	(Ghosal et al., 2012)
Plasmid: HA-Ubiquitin	Addgene	Plasmid#18712
Plasmid: SPRTN	Dr. Junjie Chen	(Ghosal et al., 2012)
Plasmid: pENTR223-USP11	Harvard PlasmID	Cat#HsCD00379313
Plasmid: pQFlag-USP7	Addgene	Plasmid#46751
Plasmid: pBluescriptR-SAMHD1	Harvard Plasmid	Cat#HsCD00333067
Plasmid: psPAX2 lentiviral packaging	Addgene	Plasmid#12260
Plasmid: pMD2.G lentiviral packaging	Addgene	Plasmid#12259
**Oligonucleotides**	**SOURCE**	**IDENTIFIER**
shRNA Targeting Sequence: USP11 #1 CTCATCTTGAAAGAGTGTG	Horizon Discovery	Cat#RHS4531-EG8237 CloneID:V2LHS_41513
shRNA Targeting Sequence: USP11 #2 ACCTCGTCATAGTAACGCT	Horizon Discovery	Cat#RHS4531-EG8237 CloneID:V3LHS_38785 7
GIPZ Non-silencing Lentiviral shRNA Control	Horizon Discovery	Cat#RHS4346
siRNA Targeting Sequence: USP11 Sense: AAGCGUUACUAUGACGAGGUAUU	Horizon Discovery	(Wiltshire et al., 2010)
siRNA Targeting Sequence: SPRTN Sense: GUCAGGAAGUUCUGGUUAAUU	Horizon Discovery	PMID: 27871365
siRNA VCPIP1: ON-TARGETplus Human VCPIP1 – SMART POOL	Horizon Discovery	Cat#L-019137-00-0005
siRNA Targeting Sequence: USP7 Sense:ACCCUUGGACAAUAUUCC	Horizon Discovery	PMID: 28325877
siRNA Control Targeting Sequence Sense: AUGAACGUGAAUUGCUCAAUU	Horizon Discovery	Cat#D-001810-01
**Primers**	**SOURCE**	**IDENTIFIER**
SPRTN ΔSprT 5′ gtggaccccacaccggacaaggaaccagagaattac 3′	This paper	N/A
SPRTN ΔSH 5′ gcccagctagtaatccctgaaacaagcaatttacct 3′	This paper	N/A
SPRTN ΔPIP 5′ gctgttagtaacagtcaccctagagtatcatttgcc 3′	This paper	N/A

SPRTN ΔUBZ<br>5′ tcatccagtcagagcaaagaaggtgacagcatcaaa 3′	This paper	N/A
SPRTN E112A 5′ accctcctgcatgcaatgatacatgcc 3′	This paper	N/A
SPRTN Y117C 5′ atgatacatgcctgtttatttgtcact 3′	This paper	N/A
USP11 C318S 5′ ctgggcaacacgtccttcatgaactcg 3′	This paper	N/A
USP11 ΔDUSP<br>5′ ccacagcacgaggagctggagctgcccaacatccag 3′	This paper	N/A
USP11 ΔUSP<br>FWD: 5′ ggggacaagtttgtacaaaaaagcag<br>gcttcatggcagtagccccgcgactg 3′<br>REV: 5′ ggggaccactttgtacaagaaagctg<br>ggtttcagatgcctggctgacc 3′	This paper	N/A
USP11 Δ426-452<br>5′ aagaagaaggagtatgtggtgatcgtggacactttc 3′	This paper	N/A
USP11 Δ453-479<br>5′ aaacggcggaacgattctcccttctgctacctcagt 3′	This paper	N/A
USP11 Δ480-505<br>5′ gtatctgtgaccttcgaccgccgcaagccagagcag 3′	This paper	N/A
USP11 Δ651-683<br>5′ aaacccaactcagatgatgctgggcccagctctgga 3′	This paper	N/A
USP11 Δ684-716<br>5′ gaccctgagccagagcagttcaccctgcagacggtg 3′	This paper	N/A
USP11 Δ717-748<br>5′ cgacgacgcaagcagctgccagagatgaagaagcgt 3′	This paper	N/A
