# Supplementary figures and images for: USP11 mediates repair of DNA–protein cross-links by deubiquitinating SPRTN metalloprotease
Source: J Biol Chem. 2021 Feb 7;296:100396. doi: 10.1016/j.jbc.2021.100396 (PMC7960550; doi:10.1016/j.jbc.2021.100396)

POLD3 Peptide Spectrum  
sp|Q15054|DPOD3\_HUMAN

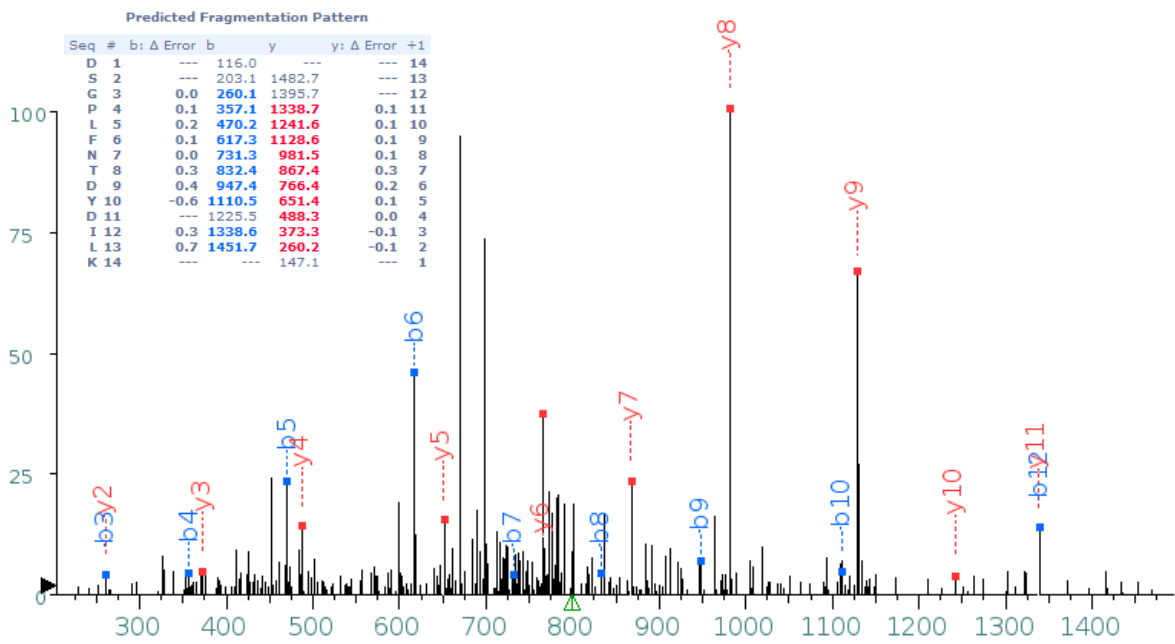

USP11 Peptide Spectrum  
sp|P51784|UBP11\_HUMAN

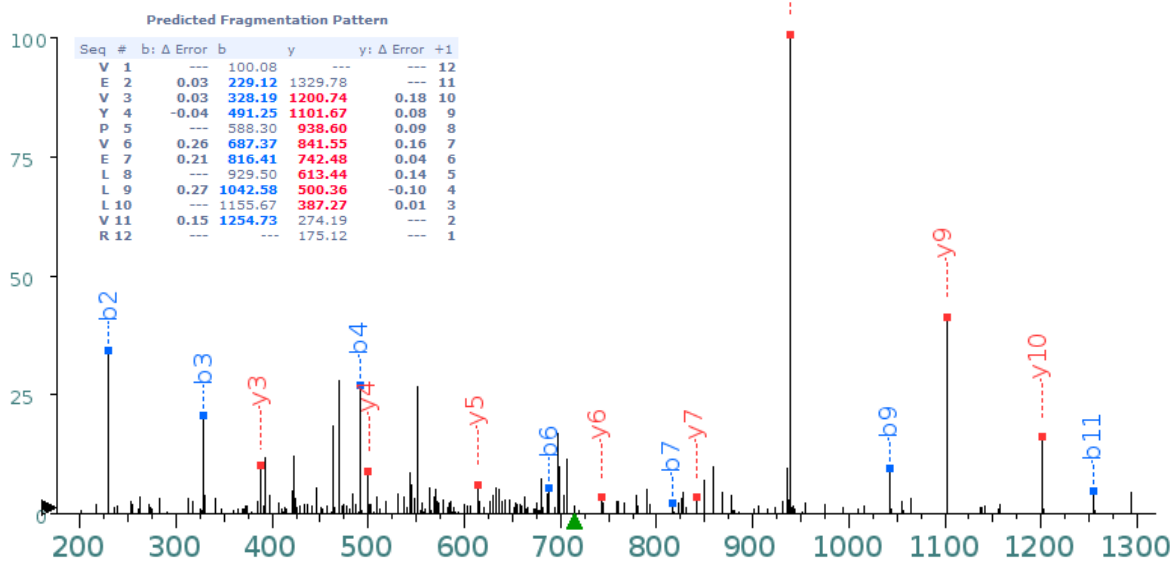

Supplement: Supplemental Data 2 [file mmc3.pdf]
